# Initial survey of blue ice areas for the recovery of old ice in the Sør Rondane mountains (Dronning Maud Land, East Antarctica)

**DOI:** 10.12688/openreseurope.21562.2

**Published:** 2026-07-23

**Authors:** Maaike Izeboud, Etienne Legrain, Veronica Tollenaar, François Pallandre, Nicolas Grosrenaud, Lisa Ardoin, Frank Pattyn, Pierre-Henri Blard, Marie-Axelle Gillain, Vinciane Debaille, Steven Goderis, Gabriel A. Pinto, Hamed Pourkhorsandi, François Fripiat, Harry Zekollari

**Affiliations:** 1 Water and Climate, Vrije Universiteit Brussel, Brussels, 1050, Belgium; 2 Laboratoire de Glaciologie, Université Libre de Bruxelles, Brussels, Belgium; 3 The French Mountain Guide Association (SNGM), Porte-de-Savoie, France; 4 International Polar Foundation, Brussels, Belgium; 5 CRPG, CNRS, Université de Lorraine, Nancy, France; 6 Laboratoire G-Time, Université Libre de Bruxelles, Brussels, Belgium; 7 Archaeology, Environmental Changes, and Geo‐Chemistry, Vrije Universiteit Brussel, Brussels, 1050, Belgium; 8 Géosciences Environnement Toulouse, IRD-CNRS-Université de Toulouse-CNES, Toulouse, France; 9 Laboratory of Hydraulics, Hydrology and Glaciology (VAW), ETH Zürich, Zurich, Switzerland

**Keywords:** Antarctica, Blue Ice Areas, Ice core, Oldest Ice, Fieldwork, Paleoclimate, Radar, Meteorites, GNSS, Measurements, Sør Rondane Mountains, UAV

## Abstract

Blue ice areas have the potential to preserve old ice near the surface, offering valuable archives of past climate beyond the range of continuous deep ice cores. Notably, million-year-old ice has been recovered at shallow depths in the Allan Hills blue ice area, in the Transantarctic Mountains. However, similar sites in other regions of Antarctica remain unexplored. The FROID project aims to identify and sample such old ice near the Belgian Princess Elisabeth Station in Dronning Maud Land, East Antarctica. This report details the first of two field expeditions, conducted during the austral summer of 2024–2025, with the goal of identifying a suitable drilling site for a shallow ice core (up to 200 m) in the Nils Larsen blue ice area. During the field campaign, we collected datasets to assess ice age, thickness and flow velocity, to determine potential and suitability for coring very old ice: (1) 10 m ice cores for absolute dating ( ^81^Kr and ^40^Ar methods), gas measurement (amongst others, δO _2_/N _2_, total air content, CO _2_, CH _4_, N _2_O) and ice fabrics; (2) surface ice samples for water stable isotope analysis (δ ^1^8O and δD) to infer paleo-temperatures and spatial patterns in isotopic variation; (3) ground-penetrating radar (GPR) surveys of bed topography and ice thickness; (4) ApRES radar profiles for vertical velocity, internal structure, and ice fabric; (5) GNSS measurements for local ablation and surface motion; and (6) Uncrewed Aerial Vehicle (UAV) tests for future meteorite recovery applications. This report outlines the field preparation, methodologies used, and challenges encountered. The results will guide site selection for the follow-up drilling campaign and can serve as a practical guide for future blue ice fieldwork.

## Plain Language Summary

Some places in Antarctica, known as blue ice areas, have the unique ability to preserve very old ice close to the surface. This ancient ice contains clues about what Earth’s climate was like in the distant past. In one of these areas, scientists have already found ice that is over a million years old. But many other blue ice areas across Antarctica have never been studied in detail.

The FROID project, led by Belgian researchers, aims to find and study these untouched sites near the Princess Elisabeth Station in East Antarctica. This report describes the first of two field expeditions, which took place in the summer of 2024–2025. The goal was to find the best spot to drill a shallow ice core (up to 200 meters deep) in the Nils Larsen blue ice area.

During the expedition, the team collected a wide range of data to better understand the age, depth, and movement of the ice. This included ice cores for dating and gas measurements, surface samples for water isotope analysis, radar surveys to map the ice and the ground beneath it, and GPS to measure ice flow and melting. We also tested the use of drones to help recover meteorites in the future.

The findings from this mission will help decide where to drill during the next field season. We also provide valuable information for planning future research in blue ice areas, which may help other scientists who are preparing fieldwork in Antarctica.

## Introduction

Blue ice areas have the potential to harbor old ice close to the surface, due to localized negative surface mass balance and emergence of old ice layers. This potential has been illustrated by the recovery of millions-year-old ice in the Allan Hills blue ice area (Transantarctic mountains) at depths of less than 200 meters. ^1^^,^^2^ The recovery of old ice in other blue ice areas scattered across the continent remains largely unexplored. Additional ice coring in blue ice areas allows to investigate past climate variations over periods not covered by continuous ice cores, as well as to provide a more detailed insight into the preservation of the climatic signal of the blue ice samples. In addition, samples from distant blue ice areas can provide regional climate variations to better constrain the past climate of the whole Antarctic continent. ^3^ To recover this old ice, two field missions were planned to explore blue ice in the surroundings of the Belgian Princess Elisabeth station. The primary goal for the first field season was to identify a site to, in the follow-up field season, extract a shallow ice core (up to 200 meters). This report details the activities carried out during the first mission.

The first mission took place in the austral summer of 2024–2025 within the framework of the **F**inding the wo **r**ld’s **o**ldest **i**ce recor **d** around the Princess Elisabeth Station (FROID) project funded by the Belgian Science Policy Office (BELSPO) and logistically supported by the International Polar Foundation (IPF). This project also benefited from additional support from Université libre de Bruxelles (ULB) Action Blanches project ( **Qu**est for world’s **o**ldest **i**ce, QUOI) for the measurement of the collected samples. During this fieldwork mission, we specifically focused on (identifying) areas where the ice thickness was <200 m, a depth that can be drilled in a single season, in order to reach the old ice located near the bedrock. Other criteria consisted of looking for a specific bedrock topography that is hypothesized to either trap old ice or force it near the surface (“cul-de-sac”), where surface velocity is less than a meter per year. The goal of this report is to document how the first fieldwork season was prepared and carried out, as well as to provide insights into the observations and some of the challenges faced in the field. As such, the goal of this report is to provide a blueprint for future Antarctic fieldwork in and around blue ice areas.

In the pre-season planning, ten blue ice patches from the Nils Larsen blue ice area were identified as potential locations of interest, using recently acquired ice penetrating radar observations (collected by the Alfred Wegener Institute (AWI) in 2023–2024), ice thickness estimates (Bedmachine ^4^, Bedmap2 ^5^) and velocity data products (MEaSUREs Phase-Based Antarctica Ice Velocity Map, Version 1 ^6^). These products provided a preliminary understanding of the area but were too sparsely distributed, too coarse and/or uncertain to capture local processes that are important to determine a drilling site. In addition, estimated terrestrial age of meteorites (unpublished) found on the blue ice in the Nils Larsen area ^7^ and on other blue ice fields in the region (Nansen, Balchenfjella and Belgica mountains) provided a first-order indication on the age of the blue ice areas at their surface (42–536 ka), based on ^36^Cl measurements performed at the Centre for Research and Teaching in Environmental Geosciences (CEREGE, Aix-en-Provence, France) by Carine Sadaka and Jérôme Gattacceca, using the ASTER accelerator mass spectrometer.

During this reconnaissance mission we gathered various samples and data to map the Nils Larsen area in detail to decide where to drill in the second mission. These include: (1) Ice cores of 10 m depth for absolute dating of the ice ( ^81^Kr; and ^40^Ar ^8^) and for gas sampling (amongst others, δO _2_/N _2_, total air content, CO _2_,CH _4_, N _2_O) to assess the preservation of the paleoclimatic signal and the absence of additional processes such as photolysis. ^9^ (2) Surface ice samples (melted) for isotope measurements (δ ^18^O _ice_ and δD), to estimate the temperature when the ice was formed, which can indicate whether it is glacial or interglacial ice and thus help to determine the ice stratigraphy. ^3^ The transects intersect with the 10 m-cores to tie the isotopes to an absolute estimate of the age and attempt to derive a surface ice age sequence by performing wiggle matching on a reference ice core record. ^3^ (3) Ground Penetrating Radar (GPR) grids for mapping the bedrock topography and ice thickness. (4) Quad-polarimetric phase Sensitive Radar (ApRES) to measure vertical velocity profiles (by combining with measurements during the next field mission), high precision bedrock depth, internal layering and ice fabric. ^10^ (5) High precision GNSS measurements of ablation stakes to determine local ablation and horizontal surface velocity after repeat measurement during next field mission, at the same location as the ApRES measurements as well as multiple other distributed locations. (6) Initial tests with an Uncrewed Aerial Vehicle to assess its potential for meteorite collection.

In this report, each type of measurement is described in more detail, including issues we encountered that might be of help to future expeditions planning similar activities.

## Overview: the team, the area and logistics

The expedition was logistically supported by the International Polar Foundation (IPF), based at the Princess Elisabeth Antarctica station in the Sør Rondane Mountains, East Antarctica (−71.95049° latitude, 23.34687° longitude), which provided field and logistical support. Since the construction of the station in 2009, the IPF has provided extensive support for fieldwork in blue ice areas (e.g., 11– 13), including visits to the Nils Larsen blue ice area as part of a meteorite recovery mission. ^14^

The 2024–2025 FROID field team spent 30 days (7/12/24 to 6/1/25) in the Nils Larsen area in a temporarily set-up basecamp at the base of the Yetnuten nunatak at ~2100 m.a.s.l (−72.34304° latitude, 22.95772° longitude). The basecamp consisted of two modified and insulated shipping containers for living/sleeping supported with solar panels and heating system, one container to store scientific equipment and one reefer container for the ice core samples. The study area is at the edge of the Antarctic plateau and subject to strong katabatic winds. Consequently, even the austral summer period is subject to harsh and windy weather conditions. Absolute temperatures ranged between −25°C and −8°C but could be experienced as low as −35°C due to windchill. The field team was able to perform field operations for 18 of the 30 days in the field due to weather conditions, which was considered better than expected (planned as 50%). For 1.5 days, operations were suspended due to limited visibility conditions, and other days operations were governed by wind conditions. These conditions can be described by sustained wind speeds (excluding gusts) in the following classes: (i) 1–2 bft (<3 m/s) allowed for comfortable working conditions, (ii) 2–3 bft (3–5 m/s) were windy but manageable days for sampling; ice coring was performed with a wind-shelter, (iii) 3–4 bft (5–7 m/s) depending on the activity, it was (not) possible to go out: static measurements came with a risk of hypothermia and some equipment (e.g. the GNSS) was subject to a too high risk of damage, (iv) >4 bft (>8 m/s) were days when we did not go out.

The 2024 Field Team consisted of six people, composed of four scientists, Maaike Izeboud, Etienne Legrain, Veronica Tollenaar and Harry Zekollari (project co-lead), and in-field support by François Pallandre (field guide) and Nicolas Grosrenaud (field technician, IPF staff ). Logistical support in Belgium was provided by Loreline Faugier (team project manager) and Lander Van Tricht (weather forecast support) from the Vrije Universiteit of Brussels, and Claire Lelouchier (administrative support) and Saïda Elmari (technical support) from the Université Libre de Bruxelles. Other FROID consortium members are François Fripiat (project co-lead), Frank Pattyn and Lisa Ardoin.

### Study area and blue ice patches

**Figure 1.**
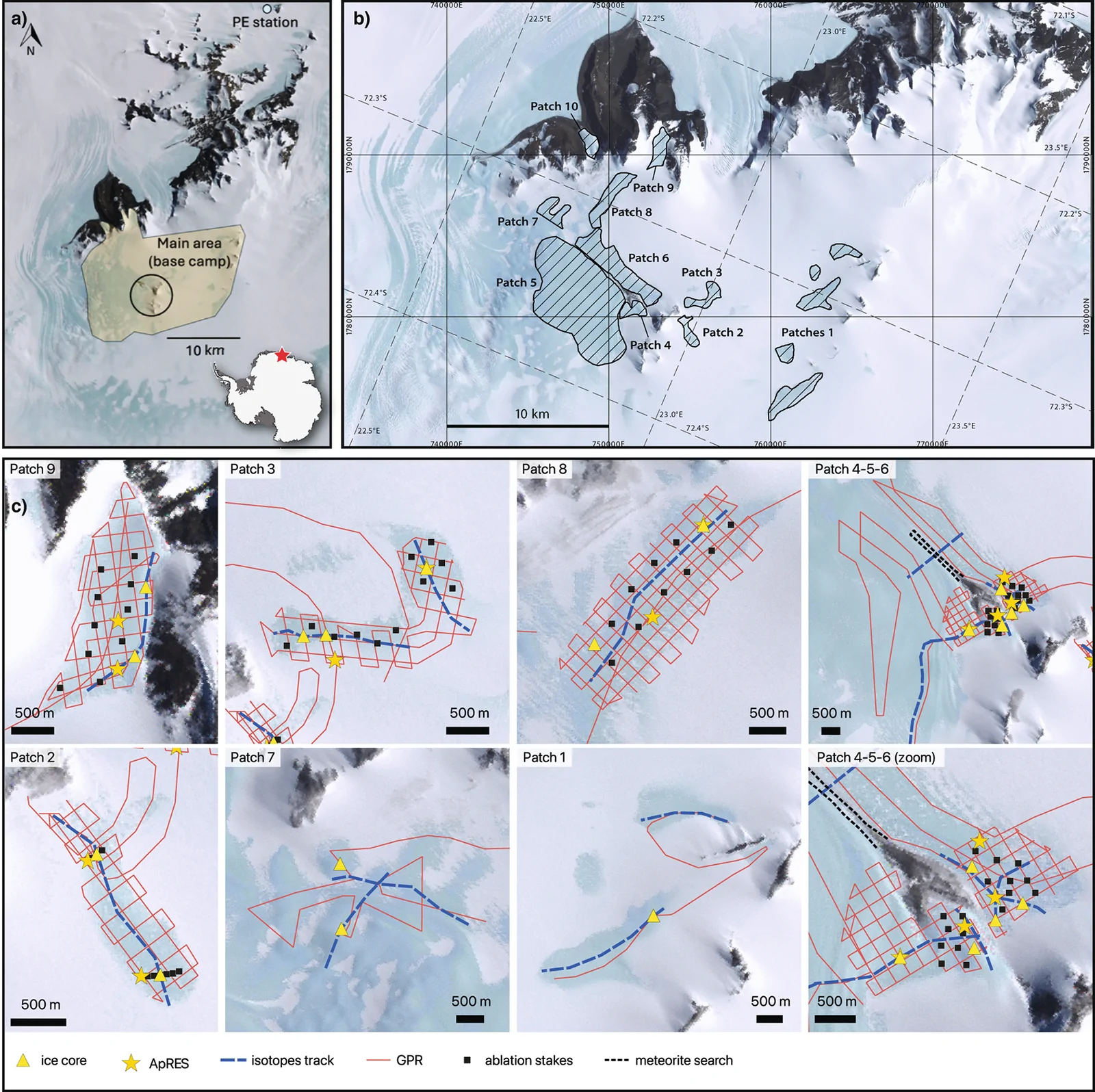
(a) Overview of campaign area, Nils Larsen, close to Princess Elisabeth (PE) Station, East Antarctica, (b) blue ice patches and their numbering, with grid overlay in both Antarctic Polar Stereographic projection (EPSG:3031, vertical and horizontal grid) and latitude longitude graticules (dashed lines) (c) tracks and locations of planned measurements within the various patches.

To establish a nomenclature for the Nils Larsen study area ( Figure 1a), we divided the exposed blue ice area into different patches, of which some directly connected to each other, while others were isolated through surface snow and/or moraines. In total, ten blue ice patches were considered as places of interest for the mission, with four patches considered to have the highest priority (Patch 9, Patch 8, Patch 4 and Patch 2; Figure 1b) due to a combination of their (low) ice flow velocity and thickness as well as suspected ice flow configuration, estimated from the data products used during pre-season planning and analysis. All patches were visited during this first field mission, except for patch 10, where the steep slopes prevented safe snowmobile navigation.

Our goal was to gather the same set of measurements at every blue ice patch to obtain a detailed overview of the Nils Larsen area:

• A reconnaissance GPR transect to estimate the general ice thickness of the patch, followed by a systematic GPR grid survey (200 m distance grid) if the reconnaissance track showed ice thicknesses lower than 200 m. Average of ~20 km per patch. Total of ~200 km collected.
• Two 10 m depth ice cores, locations determined based on on-site GPR preliminary analyses. Total of 15 cores collected.
• At least one surface ice sample transect along the direction of ice flow. Average of ~3 km per patch, sampled at 20 m distance interval. Total of 1153 samples collected.
• ApRES measurement at two locations, including ablation stakes and high-precision GNSS. Total of 12 pRES+GNSS locations.

In addition to this set of measurements per patch, we distributed additional ablation stakes in Patches 2, 3, 4, 6, 8, and 9 (additional 69 stakes), around which three surface ice samples were collected (207 samples). Furthermore, we performed a few dedicated meteorite searches in a moraine located between Patches 4, 5, and 6 (3 × 2 hours; no meteorites were collected) and placed and monitored metal cylinders (‘fake meteorites’) in Patch 3 and 5 to measure their rate of submergence.

### General approach in the field

On a typical day, the field operations were organized in two teams of two scientists working in parallel, one of the teams accompanied by the field guide and, on some days, the other by the field technician. For safety and practical reasons, both teams worked on the same patch or nearby patches, with one ‘static’ team drilling ice cores or performing ApRES measurements, and the other ‘mobile’ team performing continuous GPR measurements or surface ice sampling. This approach allowed (a) all team members to navigate to the patch with the field guide for safety (b) the field guide to assess the safety for the static team before joining the mobile team (c) to communicate via radio as there was line-of-sight on this short distance.

We used detailed daily weather forecasts from to inform decisions on the duration and type of field activities. Based on the assessed risk of hypothermia and/or risk of damaging equipment, we adjusted the planned workload accordingly. In particular, ApRES and GPR measurements were postponed on days with wind speeds of 3–4 bft, as these activities require operators to remain exposed and stationary for extended periods. Under such conditions, we instead prioritized activities less sensitive to wind exposure, including drilling (with shelter), surface water isotope sampling, and meteorite searches. GNSS measurements were performed together with ApRES measurements and therefore postponed depending on ApRES limitations.

### Ground Penetrating Radar: MALÅ Rough Terrain Antenna (RTA)

We used a MALÅ RTA of 25 MHz with an antenna distance of 6 m to measure the ice-bed interface, from which ice thickness can be derived and bed elevation is subsequently calculated by subtracting it from the surface elevation. The MALÅ system includes a connected GPS receiver (GLOBALSAT BU-353S4) which immediately co-registers coordinates (latitude, longitude) and surface elevation to each datapoint (accuracy up to 5 m). We first drove a reconnaissance transect through most blue ice patches to get a rough idea of ice thickness, which was used to assign patch priority and determine where to survey a full grid. Patches were surveyed in a grid pattern with 200 m spatial distance. The orientation of the grid was configured to align closely along and across flow based on the assumed ice flow direction. Data was collected at a 1 s interval at a driving speed of 5–10 km/h ( Table 1).

**Table 1.** Settings of MALÅ GPR.

Deployed GPR system details	
Antenna	MALÅ Rough Terrain Antenna, 25 MHz (distance Tx-Rx: 6 m)
Receiver Unit	MALÅ Professional Explorer (ProEx) control unit, Li-Ion 12 V battery
Monitor	MALÅ XV Montior, powered by Control Unit

Signal parameters	Value
Sampling frequency	252.52 MHz
Max. time window	Long
Autostacks	On
Time Window	6070.8 ns (510.00 m; 1544 smp)
Velocity	167 m/uS
Acquisition Mode	Time Triggering
Time interval	1.0 s

### Objective

We aimed to identify areas with ice thickness <200 m and favorable bed topography for trapping or containing old ice (e.g. a closed basin, also referred to as ‘cul-de-sac’) to guide the site selection for next field mission. In addition, after initial processing of the data at the basecamp, we used the thickness profiles to select suitable locations for the 10-m depth ice cores within each patch.

### Field set up

**Figure 2.**
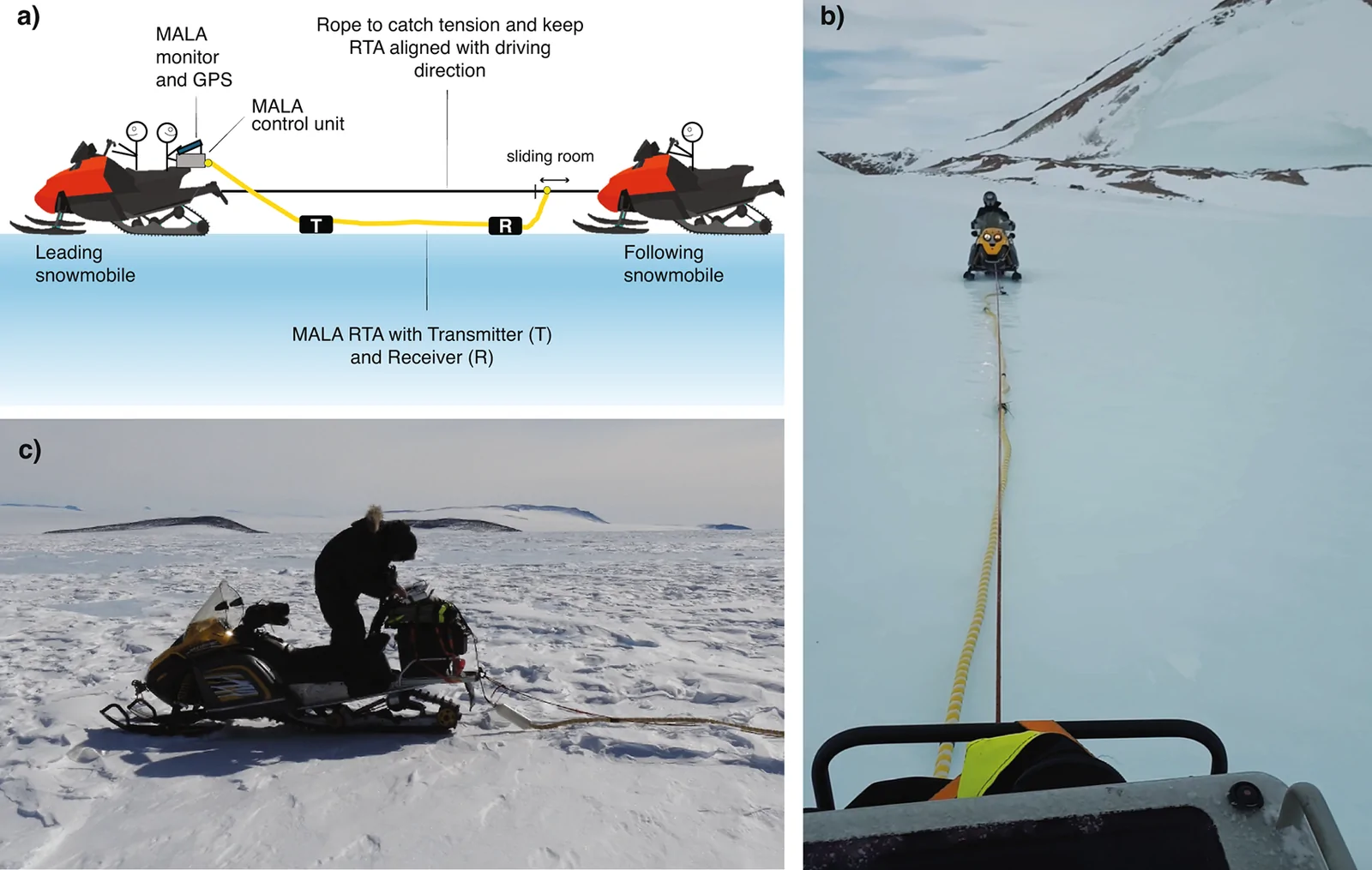
Setup of GPR. (a) Schematic, (b) the yellow snake (RTA) seen from the leading snowmobile, (c) mounting of the MALÅ system on the leading snowmobile.

The control unit and monitor were mounted on a snowmobile and the RTA (a ‘yellow snake’, ~9 m long) was pulled between two snowmobiles: the snowmobiles were attached with a rope and the end of the snake was attached to that rope to avoid tension on the RTA itself ( Figure 2). Having this configuration (with a following snowmobile pulling the end of the snake) was essential as the snake would otherwise slide forward into or under the leading snowmobile on sloped blue ice areas. The leading snowmobile carried two people: one driver and one to view the data live and troubleshoot any issues (who was sitting backwards); this last task proved to be quite essential for a smooth and efficient operation.

### In-field processing

**Figure 3.**
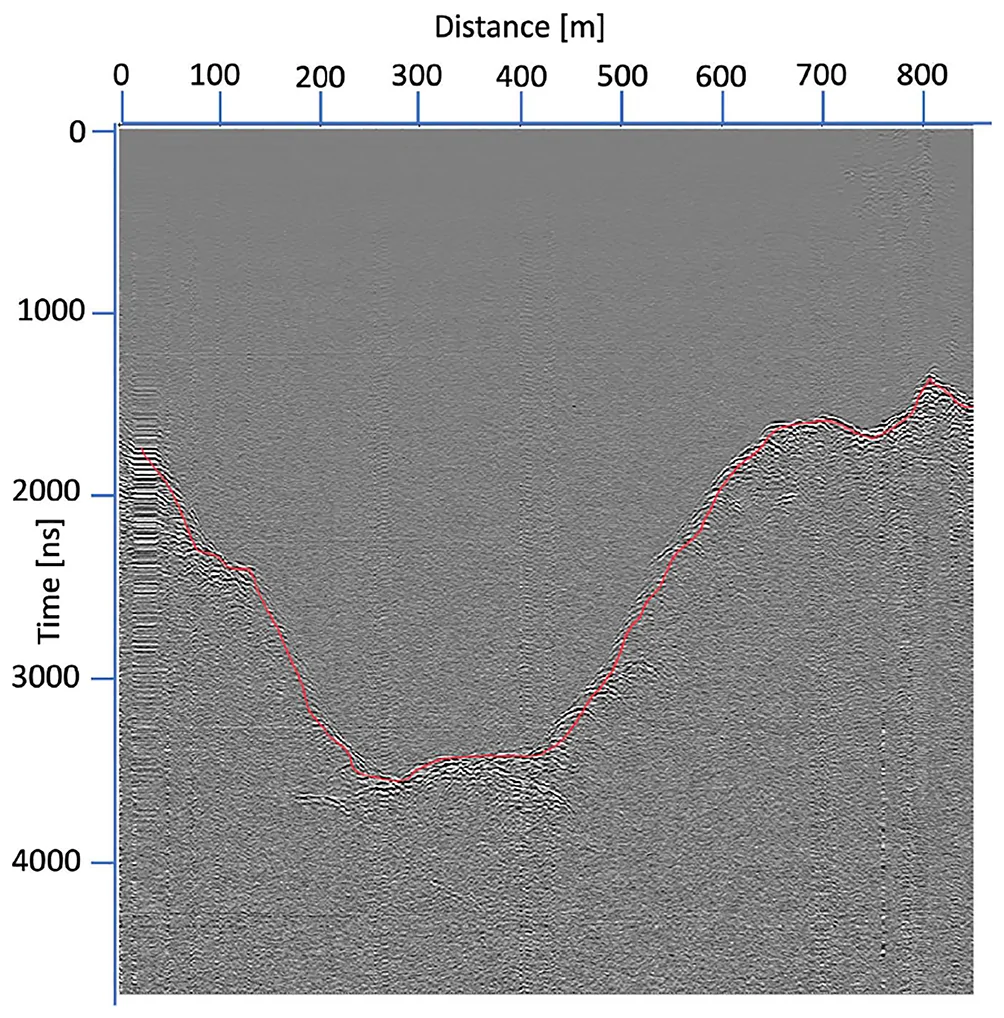
Preliminary processing of one of the MALÅ GPR tracks using ReflexW software (Patch 4). Red line shows preliminary picking of bedrock reflection in ReflexW.

In-field processing was completed at the basecamp with a simple pipeline using the ReflexW software ^15^ ( Figure 3) (signal processing; picking bedrock reflections), python (simple calculations and data aggregation) and QGIS (visualisation), ^16^ to obtain a first estimate of local ice thickness (bedrock depth). This estimate was used for determining the ice core drill locations, and as a control on the quality of the measurements.

We chose settings that yielded a clear bedrock reflection at depths of 200–250 m ( Table 1):

• Time window: 6070.8 ns (510.00 m, 1544 samples), Time interval: 1 second
• Sampling Frequency: 252.52 Hz, max time window: long, autostacks: on.

With these settings, most of the time the bedrock reflection was clearly visible up to depths of 350–450 m. We used the same settings for most of the measurements and adjusted it only a few times for deeper bedrock sections, but with limited impact on the bedrock visibility (see next section).

### Encountered challenges

**Measurement error**: this error message occurred frequently during measurement acquisition, due to disconnection of a cable(s) when driving over rough topography, such as a high sastrugi. The most common disconnections identified in the field occurred between the cable connecting the antenna and the control unit and with the cable connecting the MALÅ-integrated GPS to the monitor). Since someone was checking the monitor at all times ( Figure 2a), these issues were noticed immediately, which allowed us to stop and resolve the issue on the spot by re-attaching the cable and/or restarting the measurement.

**(In)Visible bedrock reflections**: At some stretches of the transects, the reflection was not clearly visible on the monitor during the acquisition, which was in some cases potentially related to the ice being too thick (>510 m; see Table 1 for settings). For short deep stretches, we did not adjust the settings as we crossed a narrow valley and quickly found the bedrock again.

For longer stretches without any visible bedrock reflection, in patch 5, patch 1 and patch 7, we adjusted the measuring time window/number of samples to try and retrieve the (deeper) bedrock reflection (e.g. 7844.9 ns; 1992 samples; 658.15 m). These adjustments, however, rarely yielded a clear improvement for retrieving the bedrock in these stretches of ice.

• During the Patch 5 reconnaissance line: we did not see any bedrock reflection except very close to the nunatak, up to ~250 m depth. Presumably, most of Patch 5 is deeper than 658 m.
• Patch 7: sporadic (in) visible bedrock reflection. Uncertain if this was due to variability of deep/shallow bedrock or due to other reasons.
• Patch 1: here we had sections of ice where we knew the thickness was around 250 m from preliminary airborne GPR surveys performed by AWI, but we could not see any bedrock reflections – while we should (easily) have been able to. We tried many different settings (time window, stacking, etc.) without any improvement, and concluded it was probably a hardware issue. After more troubleshooting later in the basecamp, we found that there was an issue with the transmitter, both with the wire connection (which we could replace) and the connection of the battery in the antenna (also fixed). Because of the issues in Patch 1, we are unsure if similar hardware issues were actually the cause of a ‘missing’ bedrock reflection in Patch 5 and 7, rather than the bedrock being too deep to see reflections. This issue occurred, however, at the very end of our campaign, leaving us no opportunity to repeat measurements in any of the patches to confirm or reject this hypothesis.

**Vertical interference lines in data:** occured sometimes, when autostacking was off. If settings were adjusted, autostacking was switched off automatically and had to be set to ‘on’ again.

**Fuel consumption**: by driving with two people on a snowmobile (300 cc) at 5–10 km/h speed, fuel consumption was estimated to be close to 30 liters per 100 km.

**Interference with radio**: the signal of the MALÅ measurements interfered with the handheld radios, blocking communication during active measurements. This was taken into account when planning our activities for the day, and frequent breaks were taken to check-in between the two teams.

**Duration of field activity**: we initially expected to complete ~10 km of transect per day, but often reached ~20 km. On colder or windier days (> 3 bft), this was only possible when shelter was available along the route or by returning to basecamp halfway.

**Equipment degradation**: due to the hard but rough surface the system endured a lot of (vibrational) impact. We had spare equipment of batteries, fibre optic cable and ethernet cables, all of which were used by the end of the campaign.

## 10 m-depth ice cores

Insights from the MALÅ GPR data were used to determine two drilling locations per blue ice patch where the ice thickness was around 200 m or less. We collected a total of 15 cores of ~10 m depth, for which the lowest 3 m (at depth of approx. 7–10 m) were kept and transported back to Belgium for analysis. Additionally, for two ice cores, the full 10-m were collected. The drilling was performed with a Metabo b7532/4 powered with a field generator and attached to a 1 m-long core barrel that produces 8 cm-diameter core. The core barrel was handcrafted by the technical team at the Université libre de Bruxelles, along with the cutting blades used to drill the ice, and had an outer diameter of 8 mm and was made of stainless steel.

### Objective

Collect ice samples for:

(1) absolute dating (Krypton ( ^81^Kr) and Argon ( ^40^Ar) ^8^) of the ice, to constrain the ice age at a depth of around 7–10 m. We did not collect the ice of the first seven meters to avoid the influence of surface processes such as solar radiation and large seasonal temperature variations, which could alter the gas content and compromise the dating. ^17^

(2) gas sampling (amongst others, δO _2_/N _2_, total air content, CO _2_, CH _4_, N _2_O) of the two full-length 10-m ice cores to assess the preservation of the paleoclimatic signal and evaluate potential alterations from in-situ production, consumption, and photolysis.

### Field execution

Our drilling operations were typically carried out by a team of three, as additional personnel provided limited benefit, while working with only two significantly increased the physical effort and time required. On average, we drilled two 10-m depth ice cores per day, with an approximate duration of 3 hours per core depending on the ice quality.

Our sampling strategy differed between objectives. For objective 1 (dating), only the bottom 3 meters was retained. The upper 7 meters were discared in the field, which reduced logging and wrapping time. After extraction, the retained core sections were wrapped and stored in styrofoam boxes placed in the shade. At basecamp, samples were transferred to a refigerated container (−15 to −20 °C) to prevent melting and limit gas diffusion. From each retained 3-m ice core, we selected ~30 cm (>1.3 kg) of ice for argon dating and ~ 30 cm (>1.3 kg) of ice for krypton dating, with the remaining 2.4 m ice preserved as back-up for dating and for potential future measurements.

For objective 2, we preserved and documented the full 10-m core length, which required an entire field day per core. For these cores, and a subset of the other cores, we additionally measured the ice temperature at various depths ( Table 2), by drilling a small hole in the ice core and placing a thermometer in it.

**Table 2.** Ice core temperatures measured in the field directly after extraction of the core piece. ^16^

Core	Depth (cm)	Temperature (°C)		Core	Depth (cm)	Temperature (°C)
**P8C2**	5	−6,3		**P4C2**	85	−11,4
(~2150 m elev)	82	−11,5		(2100 m elev.)	124	−13,3
	92	−10,7			166	−14,3
	147	−13,5			247	−17,3
	176	−15,5			285	−18,4
	211	−16,2			333	−18,5
	254	−19			363	−19,3
	293	−20,4			395	−20,4
	355	−20,4			450	−18,7
	406,5	−21,8			467	−20,2
	447	−23,3			504	−20,5
	466	−23,1			545	−21,1
	516	−22,9			590	−21,4
	555	−20,8			620	−21,1
	605	−22,5			672	−21,1
	693	−22,7			700	−21,4
					740	−22,1
**P6C2**	5	−9,5			785	−22,2
(~2133 m elev)	70	−10,1			814	−21,0
	85	−10,5			860	−21,2
	150	−12,8			893	−21,5
	200	−13,7				
				**P7C1**	735	−18,0
**P8C3**	5	−10,6		(~1987 m elev)	940	−17,6
(2099 m elev.)	75	−10,7			1040	−17,5
	155	−13,7				
	225	−16		**P1C1**	10	−9,3
	265	−18,4		(2214 m elev.)	100	−10,5
	315	−19,5			200	−16,0
	370	−20,8			250	−17,2
	455	−22,5			300	−19,7
	545	−22,5			420	−19,8
	645	−22,5			520	−23,7
	725	−22,3			600	−23,6
	805	−22			650	−24,0
	885	−23,2			690	−23,8
	955	−23			730	−24,1
	1005	−23,2				

**Figure 4.**
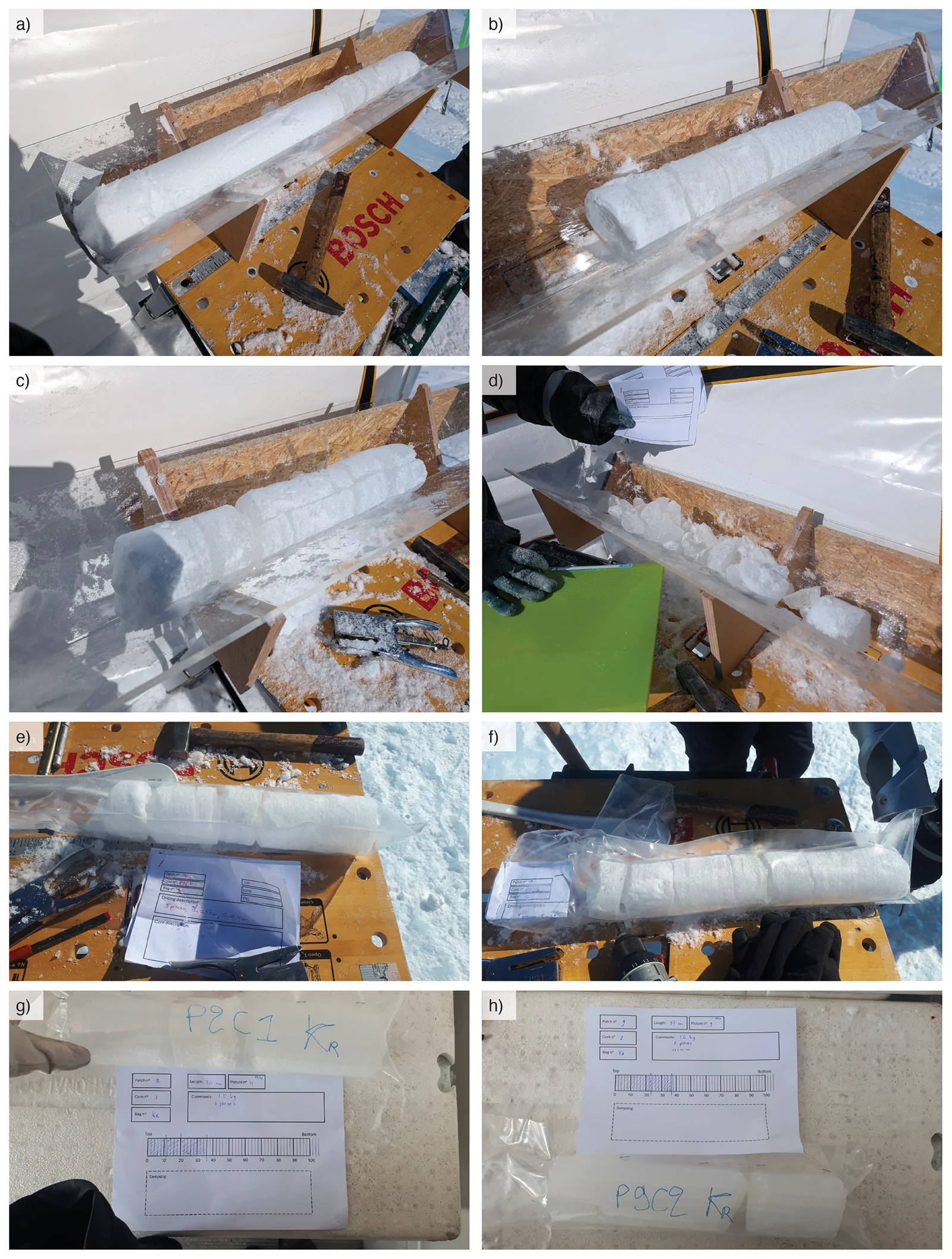
Ice coring examples of (a) how the barrel is filled largely with tiny ice chips and only ~ 20–30 cm of proper ice core, (b) a ‘good’ ice core sample with pieces of 10–20 cm, (c) ice core with vertical fracture in the ice, (d) extremely bad piece of retrieved core (not preserved), (e) logging of core sample, including some very narrow disks (f ) a packed core sample ready to bring back to the basecamp. The worktable has a width of 1 m. (g) and (h) examples of Krypton (Kr) sample selection and packing.

On average, we retrieved around 40–50 cm of ice samples per fully drilled barrel due to the accumulation of ice chips within the barrel during drilling ( Figure 4a). The ice core was usually in pieces of 5–15 cm, sometimes up to 30 cm and sometimes completely broken into very narrow disks; see Figure 4b-f. For the Ar and Kr dating, we selected the largest intact pieces of ice until a minimum of 1.3 kg of ice was selected, which corresponds to~30 cm of ice core – in many cases this was reached by combining two ~15 cm pieces ( Figure 4g-h).

The ice core samples were packed and transported in styrofoam boxes (one 3-m core sample collection per box), filled with snow or ice chips to support and insulate the samples.

### Encountered challenges

**Ice quality:** Our main concern was the lack of intact ice: the ice core pieces we collected were almost never longer than 20 cm and often smaller. This is a common feature of blue ice that contains many fractures especially near the surface. In addition, the use of a mobile drill (Metabo b7532/4) likely limits the quality of the ice core. The quality of the ice was unpredictable before drilling, as the quality of the ice at the surface seemed to not be correlated with the quality of the ice at depth. Most often the quality of the ice varied in depth, with a few stretches of fragmented ice occurring. For the absolute dating, the ice samples need to be as long as possible and without fractures. Therefore, we tried to have sufficient quality pieces of 10–20 cm before stopping to drill and otherwise extended the drilling until 11 or 12 m depth. One core (in P8) was of such bad quality that we drilled a whole new core in a nearby location. We only consistently logged the temperature of the ice for the two full-depth cores (see Table 2), as often the drilling of the small hole for the thermometer would break the sample in smaller pieces.

Challenges with the **equipment**:

• We were prepared that the drill blades would break in the blue ice, as detailed by other field experiences (communication with S. Shackleton and J. Peterson ^18^^,^^19^), and brought many spares. None of our blades broke, presumed due to the absence of rocks or sediment particles into the ice. Drilling sites were also selected for the absence of too many rocks at the surface.
• The electrical cable of the drill head was broken at one point and was repaired by the field technician. We brought a spare drill head and so did not have any delay due to required repairing of the cable.
• The plexiglass of the V-shapes ( Figure 4) used for in-field processing was quite fragile and broke for two of the three V-shapes we brought in the field. It was also possible to process the core without a V-shape by using the gutter in our worktable.
• The springs at the bottom of the barrel (‘core-catches’), which prevent the core from falling out, occasionally malfunctioned. This was caused either by ice chips, snow, or melt/refreeze processes obstructing their movement (preventable by clearing them before lowering), or due to reduced spring strength (resolved by replacing the part). When this occurred, part of the core sample could drop out of the barrel when lifting the system out of the hole, leading to damage during the next drill section. To minimize this risk, we adopted a consistent retrieval procedure after each drilling section: (1) slightly lift the drill to break the core free from the ice, (2) gently push the system downward to reposition any segment of the core back into the barrel behind the springs, and (3) start lifting the whole system out of the hole to retrieve the core sample.
• The connection of the metal extension rods was sometimes difficult, where they would not slide over each other, possibly due to temperature differences or due to presence of snow/water/ice. This difficulty increased with temperature, due to the presence of liquid water during the warmest days of the mission. The bad connection between these pieces increased the risk of dropping the whole system in the hole as one person is holding the whole system while another is attaching the next extension. The whole system can be quite heavy (25 kg at 10 m depth) and this step should not take too long, and the extensions can be attached wrongly as a result. This happened to us twice and twice the barrel was left behind in the hole when we were lifting the system out (after which we could recover the barrel, see below).
• We calibrated the blades of the drill to adapt the settings to blue ice (versus firn) as we could initially clearly see drill-lines on the side of the core samples. The optimal setting was 1.0 mm (the blades protrude 1.0 mm from the top of the core barrel).
• The staplers for the on-site logging suffer from the cold and windy conditions: as a result, they malfunctioned during the second half of the mission, complicating the logging of the cores. We brought two staplers, but both were unreliable by the end of the mission. We advise to bring a stapler that has easy access to the staple-area and/or can be opened in order to make small repairs.

**Figure 5.**
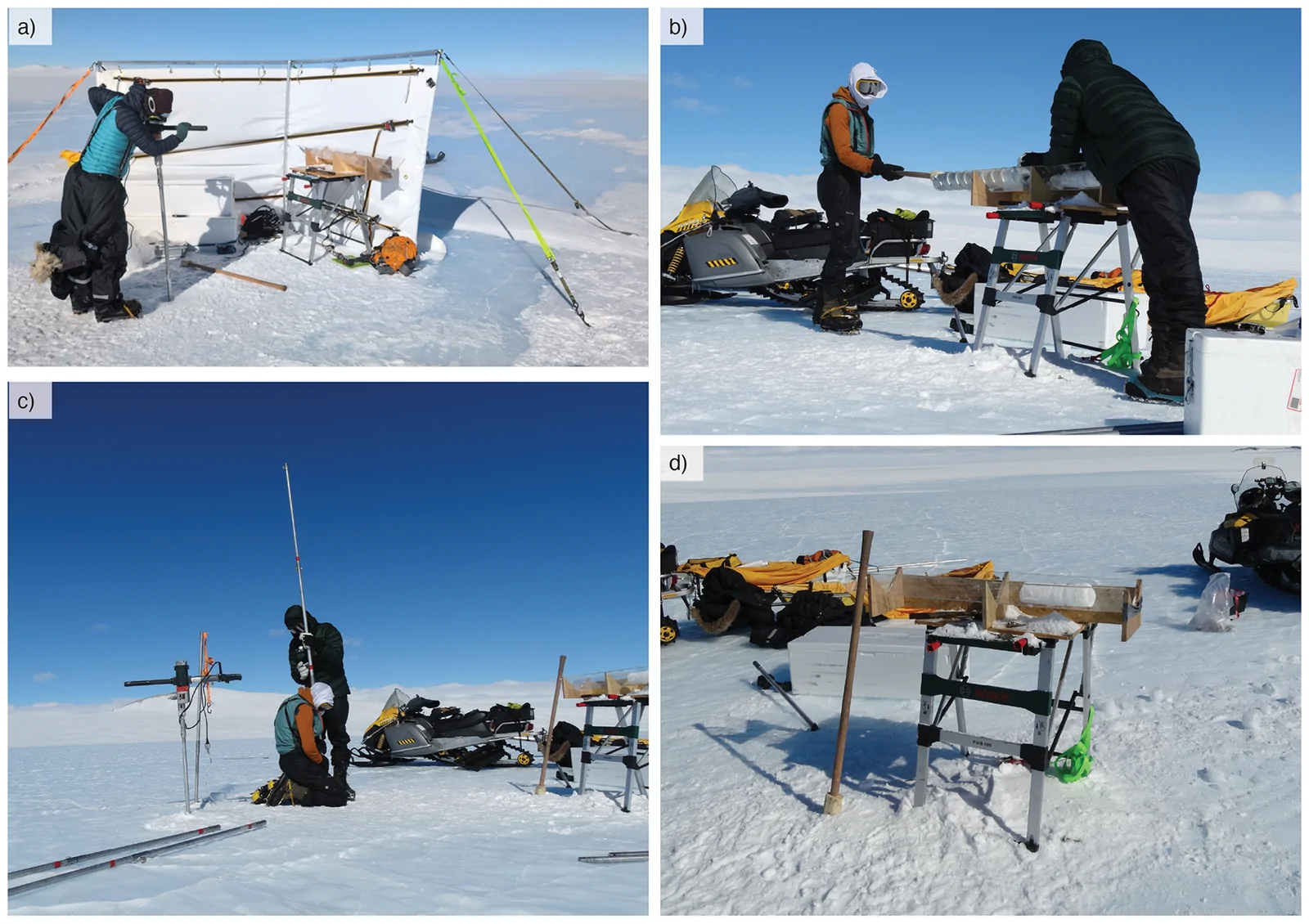
Ice coring set-up. (a) With wind shelter on days with wind ≥2bft, anchored with ice screws. (b) Pushing the core sample out of the barrel with wooden fitted head. (c) Drillhead placed on one (unused) extension during extracting/lowering of the barrel to keep drillhead free from snow/ice. Extensions usually added/removed by the pair for faster execution. (d) logging set-up with worktable, V-shape, two white boxes for sample transport and the wooden stick.

Challenges in **execution**:

• Difficulty getting the **ice core out of the barrel** (using the fitted wooden tool, Figure 5b,d): A lot of tiny ice chips (looked like snow) were created when drilling the blue ice that fell in the barrel (which is open at the top) and got compressed. This snow-like consistency created a lot of friction and made it difficult to push the sample out of the barrel. This issue was mostly solved removing as much snow from the top of the barrel as possible (using a ladle). The moment the barrel is full can be felt during drilling by a change in the pressure needed to push down, and we stopped immediately each time to limit the compression (and potential fracturing of the ice). As a result, the ice cores were at most 50 cm per barrel. During two of the warmest days (−8 to −6 °C), it was difficult to get the ice core out of the barrel. Due to the large temperature difference between the surface and within the hole, some ice particles in the barrel would melt and, when drilling the next section, would refreeze – sticking the new core firmly in the barrel. The refreezing could be reduced by drying the barrel before lowering it back in the hole, but more effective would be to wait for colder conditions.
• **Barrel stuck in drill hole**: this occurred mostly due to tiny ice chips filling the hole above the barrel and/or not drilling perfectly vertical, creating a barrier for pulling the barrel out. It was challenging to pull the system out when it was stuck, as there is no efficient way to apply large upward forces and/or to pull with many people together. The solution was to create an upward momentum, by slowly lowering the system down and quickly pulling upwards, in short successive movements, to break through the barrier. This could be executed by one person after the other for short intervals, which was more effective than pulling with multiple people at once.
• **Recovering a dropped barrel**. This happened two times. One time we had retrieved only one extension and the remaining 6–7 m plus barrel remained in the hole. We were able to recover the remaining equipment by pushing the last extension back on top of the one visible in the hole and lifting just based on the friction between the extensions (without the connecting pin). Another time we retrieved all but one extension and were able to ‘fish’ for the barrel at ~4 m deep by using a hook on a rope. The barrel dropped both times due to faulty connections of the extension rods, which should always be checked thoroughly before lowering the system, to prevent this from occuring. Connection pins should be kept dry.

**Challenges with wind conditions.** A wind shelter protected the drilling team from the wind during most ice coring days ( Figure 5a). Not only did the shelter protect the operators from windchill, but it was necessary for the execution of the handling and wrapping of the core samples (paper, pencil and plastic wrapping for samples are easily blown away). If winds were at ~2 bft or higher we set up the shelter, which took about 20–30 minutes. An added benefit of the shelter was that the 2 ^nd^ team performing MALÅ GPR grids could also use the shelter to warm up.

## Surface water isotope samples

### Objective

Collect surface ice samples along transects for ice δ ^18^O and δD measurements in the lab. These measurements will give an indication about the past surface air temperature, and may provide insights into ice stratigraphy by performing wiggle matching to a reference surface air temperature record. ^3^ As the transects of samples intersect the 10 m depth cores, their relative dating can be confirmed and pinpointed, and potentially narrow down the uncertainties of the absolute dating. Moreover, deviation of both ice δ ^18^O and δD from the meteoric water line make it possible to determine whether the ice formed through snow compaction and/or melt–refreezing processes.

The samples are being analyzed with a PICARRO L 2130-i cavity ring-down spectrometer (CRDS) at the Archaeology, Environmental Changes, and Geo-Chemistry lab of the Vrije Universiteit Brussel (VUB). Measurements are reference to Vienna Standard Mean Ocean Water (VSMOW) using in-house standards waters, bracketing the δ ^18^O and δD values (i.e., δ ^18^O (‰) = ( ^18^O/ ^16^O) _sample_/( ^18^O/ ^16^O) _SMOW_– 1; δD (‰) = (D/H) _sample_/(D/H) _SMOW_– 1). The measurement protocol was similar as during the 2022 and 2023–24 fieldwork missions of the Instituto Antártico Chileno (INACH) to Union Glacier, Ellsworth Mountains and previously applied in the PEA area in 1988–89, 2012–13, 2018–19 and 2019–20 seasons. ^3^ To avoid influence of surface melt on the samples, the ice is taken below the uppermost surface layer by removing the first few cm (approximately 8–10 cm) of ice and then taking the next 8–10 cm of ice as the actual sample.

Ice samples were taken on transects approximately along ice flow lines to capture the age gradient of the blue ice patch. Additionally, one across-flow transect was made from P5 to P6 to cross the moraine and sample the junction of two (presumably) merging ice flows.

### Field sampling protocol

**Figure 6.**
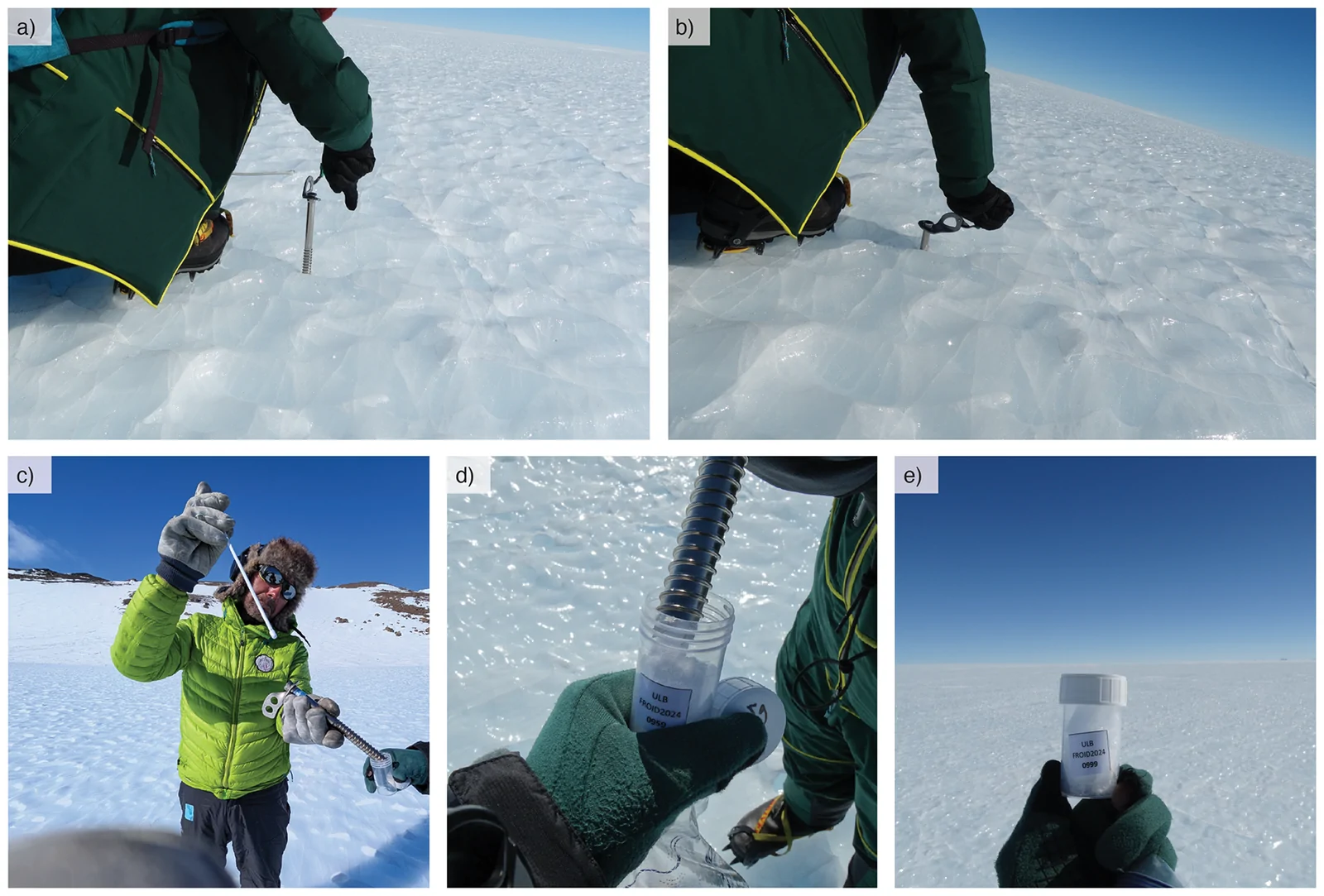
Surface ice sampling. (a–b) Person 1 using ice screw to take ice sample at few centimeter depth (c–d) Person 2 providing bottle with correct sample number, person 1 emptying screw into bottle using a stick, followed by Person 2 marking the GPS location. (e) filled sample bottle.

A transect line of approximately 3 km was planned in each blue ice area ( Figure 1 and Figure 6), with samples collected at ~20 m intervasl. Transects were conducted on foot, using distant reference points (e.g. a mountain top or parked snowmobile) to walk a straight line. One person walked to each sample spot, approximating the distance between sampling points by counting steps, and would collect the ice sample with an ice screw. A second person followed, prepared the polypropylene (PP) bottle to deposit the ice sample (60 ml, 33 mm diameter, 70 mm height, brand: VWR ^®^), kept track of the correct bottle number for each sample, and marked the sample location with a handheld GPS. When a third person was available, two would be leapfrogging and collecting the ice samples, while the third would be following with the bottles and the GPS. The snowmobiles were parked at the start and end of the transect.

All bottles were labelled before departure to Antarctica, taking approximately ten hours of work with two people. Care was taken to ease the field handling of all bottles, making sure each marked GPS waypoint number could be associated with the right sample. Each transect was sampled in ascending order, and finished in multitudes of ten. For example, if we would complete the planned transect in a patch at sample bottle #0432 we would extend the transect until #0440.

**Taking the samples:** we explored two options to take the ice samples: (a) with a chisel, hacking and crushing the first few cm of ice and removing this by hand (relatively easy to wipe to the side), then hacking and crushing the next few cm of ice to take as sample, and (b) with a long ice screw (hollow inside), screwing once halfway into the ice and removing the contents, then screwing a second time fully, using these contents as sample ( Figure 6).

We preferred method (b), as it was a very consistent method and relatively fast. The main downside was that the sample bottles were not filled to the top, which could be more easily achieved when using the chisel. As we were taking hundreds of samples, we gave priority to speed and ice quality over volume per sample. Moreover, isotopic measurements on samples obtained in previous field missions indicated that a isotope fractionation, possibly occurring due to sample - air contacts within the bottles, was negligible on the measured isotopic ratios. ^3^ We used high-quality, stainless steel ice screws with a free-rotating handle (Petzl screws used for ice climbing of ~20 cm length), which proved essential for efficient sampling.

### In-field processing

**Figure 7.**
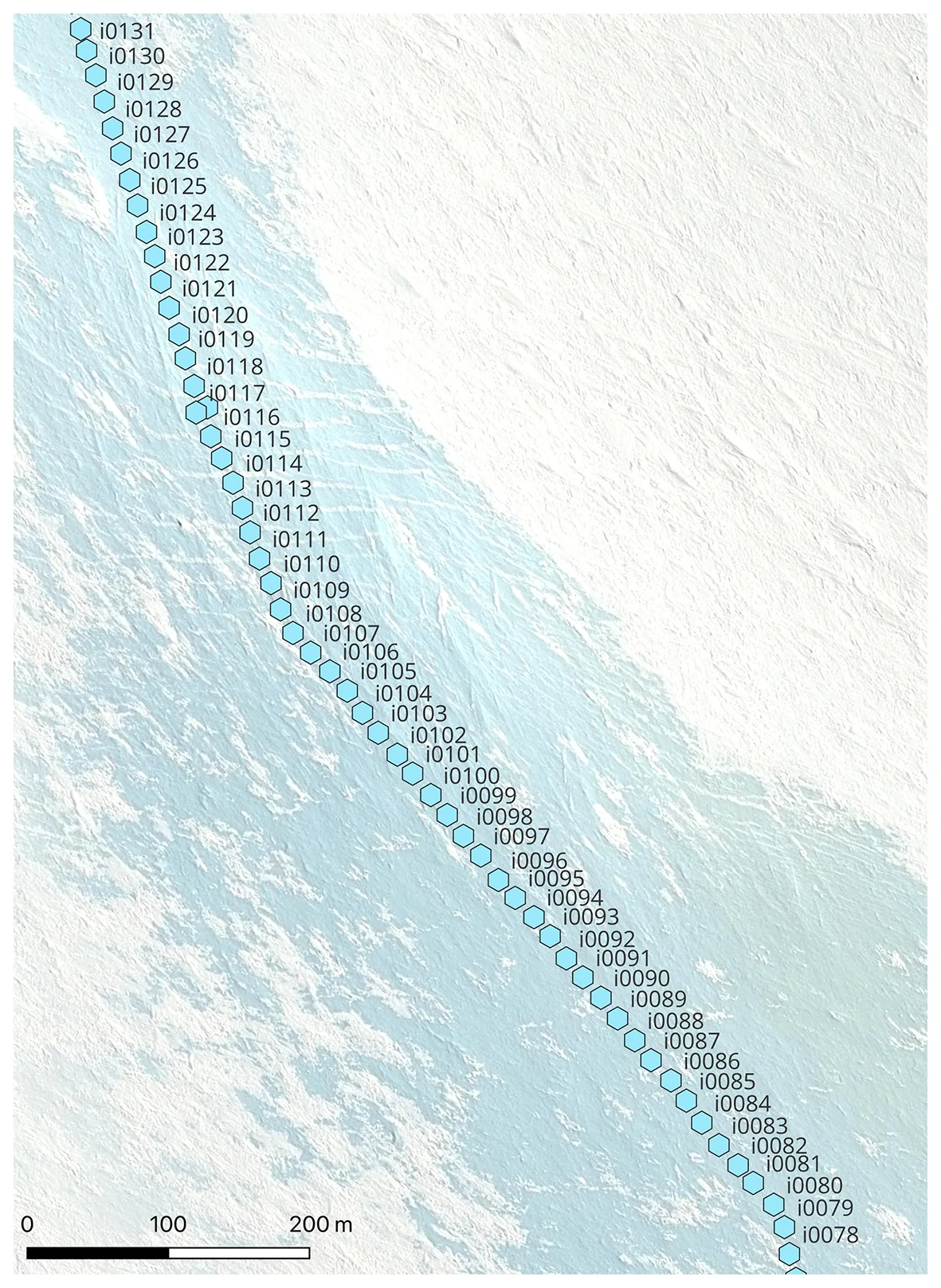
Close up of registered sample locations (after renaming of gps waypoints) in Patch 2.

Each evening upon returning to camp after sampling, all recorded GPS waypoints were manually renamed to match the corresponding sample numbers on the bottles ( Figure 7). This process was carried out using the Garmin BaseCamp software.

We did not process the samples on site. To preserve the quality of the samples until their measurement and reduce the risk of isotopic fractionation, ^20^ it is best to melt the samples once and avoid multiple melt-freeze cycles of the ice. We melted and stored the samples in the basecamp within our (above 0 °C) sleeping containers. Once melted, we closed every bottle more tightly (they could be loose due to vibrations on the snowmobile or just due to poor initial tightening due to working with thick gloves, or ice chips in between the lid closure). We then taped the bottles with duct tape to prevent leakage of air. This activity was time-consuming and usually executed during “bad weather days”.

## ApRES measurements

We deployed a Phase-sensitive radar (ApRES) on two locations on each blue ice patch ( Figure 1). In some cases, these locations coincided with the ice core site, while in others they were selected for their added value in providing bedrock information. ApRES phase-sensitive radar is a low-power, light-weight instrument developed in a collaboration between the British Antarctic Survey and University College London. It is a 200–400 MHz FMCW radar, with a 1-second chirp (nominal power of 20 dBm, or 100 mW), run by controller in yellow Pelican Box, powered by a snowmobile battery (Pb, 12 V/18 Ah). ApRES measurements are ideally conducted on ice thicker than 200 meters to ensure reliable signal returns. The choice of location was therefore a trade-off between the distance to the ice core and the thickness of the ice, which was informed by preliminary MALÅ radar results.

We used consistent calibration settings across most sites and performed initial data processing in the camp for a data quality assessment. At four locations, data quality was insufficient, and the these four sites were subsequently remeasured with different settings after recalibration (detailed below).

### Objective

Obtain high precision radar profiles that will be repeated in the next field mission to determine vertical ice flow velocity profile (emergence velocity), as well as to map the internal structure of the ice and provide independent bedrock depth measurement to be combined with the MALÅ profiles.

### Field set up and measurement settings

ApRES measurements were taken in quad-polarimetric configuration, using two antennas (metal grid-like structures) that were rotated to cover HH, HV, VV, VH configurations. The distance between the antennas was seven meters, and antennas were oriented to their connecting line to approximately be along flow. The transmitter antenna was consistently put upstream of the receiver antenna, and the orientation between the antennas was noted. We marked the location of the two antennas by installing bamboo stakes, which were also used for ablation measurements (see section “GNSS measurements and ablation stakes”). These stakes are essential for the repeat measurement during the next field mission, which needs to be performed over the same ice layers to detect a vertical velocity profile. For the latter, only the HH measurements are needed.

**Figure 8.**
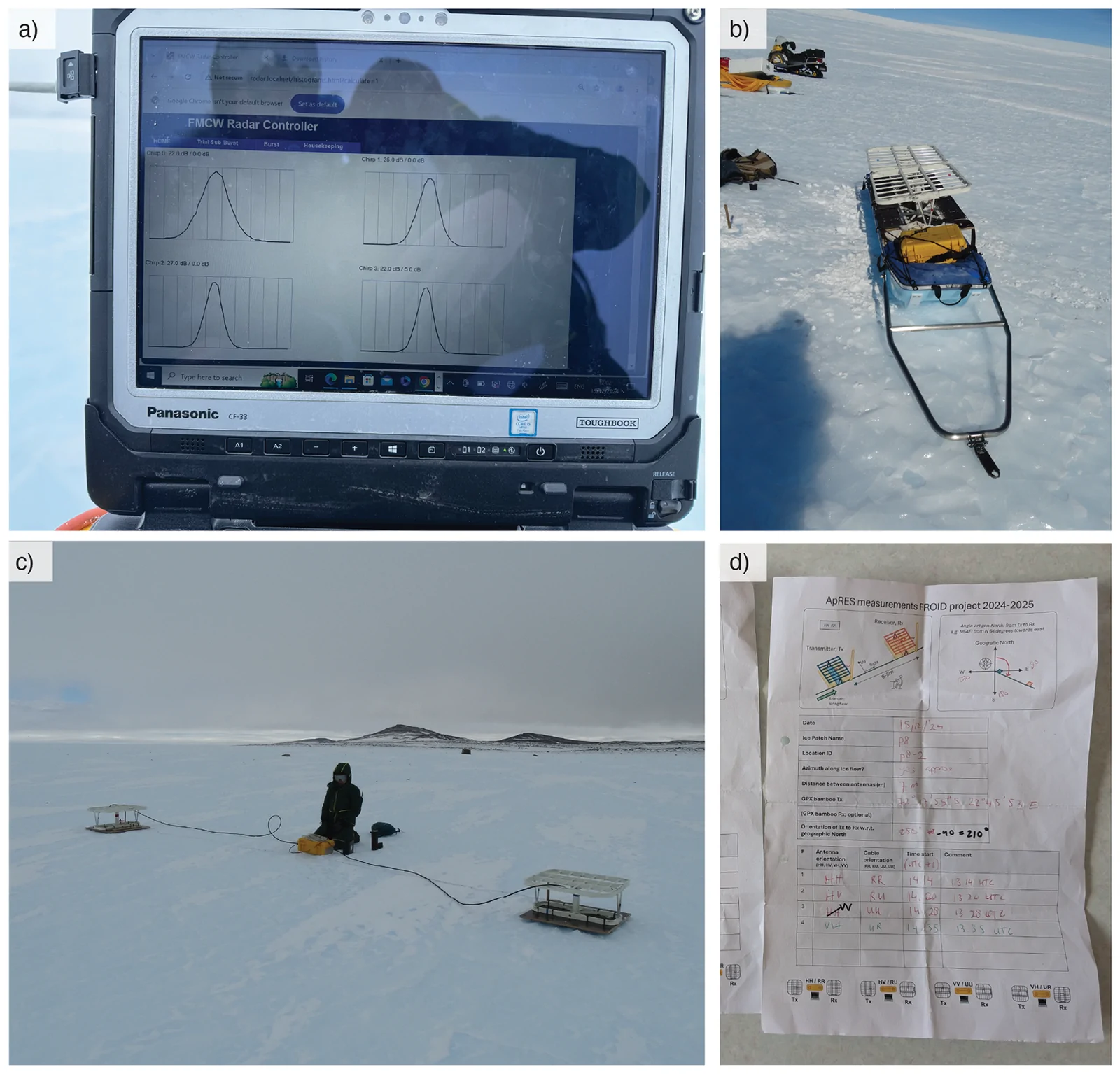
ApRES (a) In-field calibration, testing different attenuator/gain combinations. From this, we chose 25/0 (top right curve: narrow bell-shape with smooth peak). (b) Equipment on a sled to transport to/from field location. Includes: 2 antenna’s, 1 receiver and 1 transmitter cable, yellow box with operating equipment, and a chisel. Optionally: the Toughbook laptop. (c) Measurement on Patch 8, VH_UR configuration. Transmitter at Vertical/Up position; receiver at Horizontal/Right position. (d) Example log of measurements at one location. Both antenna orientation and cable direction are denoted to prevent writing/logging errors.

The attenuator settings were first calibrated in the field by testing multiple configurations of attenuator/gain and kept the same for 8 (of 12) of the locations ( Figure 8a). Attenuator/Gain: 25/0. For the 4 locations where these settings were not providing good-quality results, the settings were 22/0 after recalibration.

The snowmobiles were always parked at >10 m distance to avoid interference of large metal objects with the signal. The antennas were mounted on large wooden plates for stability; the point of each antenna was placed in a small hole in the ice made by a chisel. Furthermore, we did not use radio communication during active measurement times (four intervals of ~6 min). Since the MALÅ signal interfered with the handheld radios, we assumed the radio signals could interfere with the (more sensitive) ApRES signal.

### Encountered challenges

**Figure 9.**
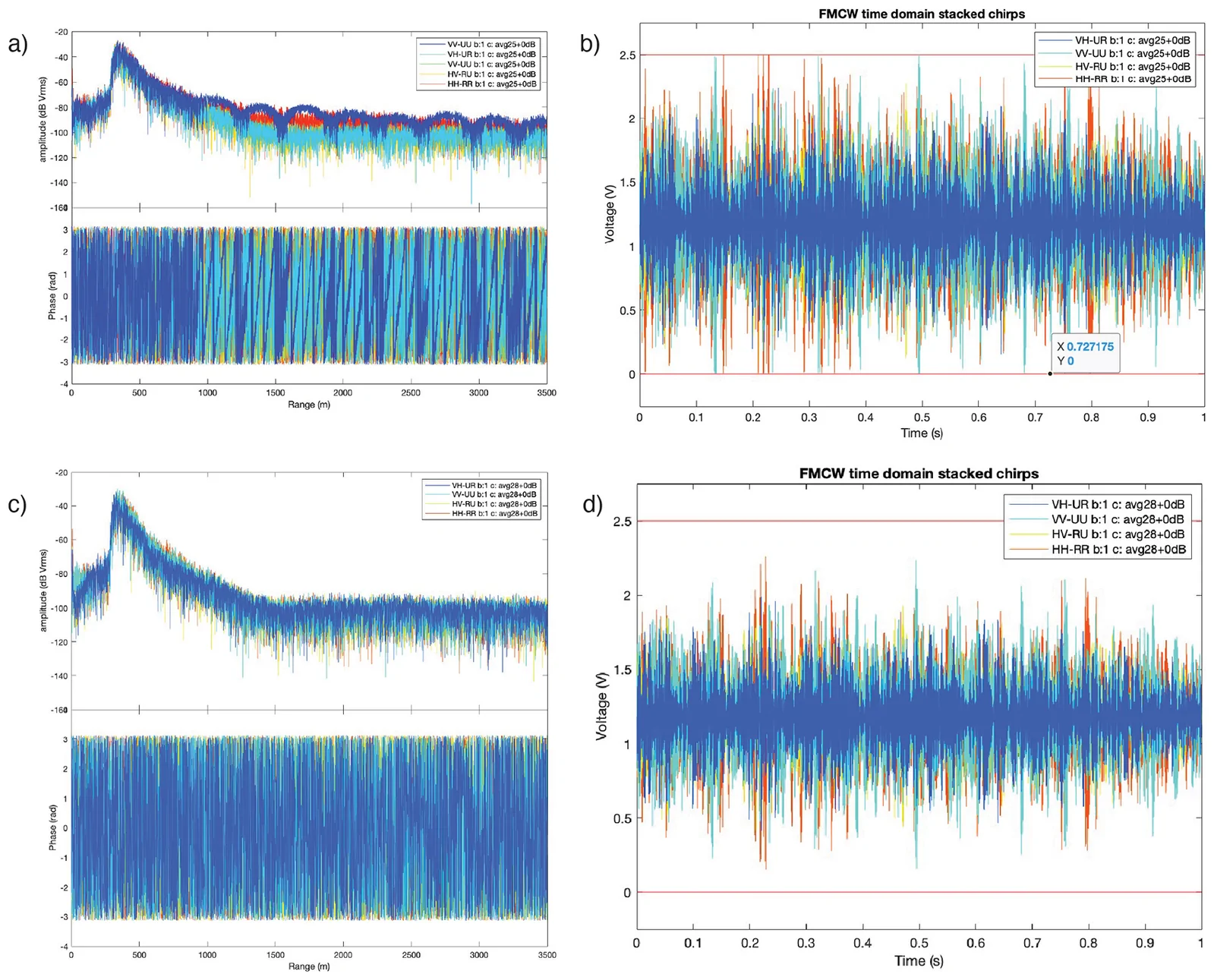
Initial ApRES processing, example of P2–1 location. (a–b) Measurements with signal of insufficient quality obtained with Attenuator/Gain: 25/0 settings. Showing arch-like features in Power spectrum (a) and voltage range in time domain (b) close to allowed bounds, indicating saturation of the signal. (c–d) Measurements at the same location with updated Attenuator/Gain: 22/0 settings, power spectrum and time domain as expected. The high peak at about 300 meters indicates the bedrock reflection.

Firstly, we expected that one configuration would be good for all locations, but the single setting yielded an oversaturated signal at some locations ( Figure 9). Performing an initial data processing step at basecamp proved essential for quality control, as it allowed us to identify issues and carry out recalibration if needed. An alternative would be to bring a computer into the field, though this is often challenging due to weather conditions. Secondly, at some point the config.ini file would not upload correctly to the SD card of the pRES via *radar.localnet* – failing to start measurements as a result. This took half a day to troubleshoot and was only solved with a workaround, by manually moving the config file to the SD card. Lastly, some elements in the ApRES box detached during transport due to strong vibrations when driving over blue ice. In the case of a detachment, all elements were intact and we were able to reattach easily. Nevertheless, future missions should avoid the risk of damaging these elements by not transporting the box in the sled, as electronical repairs are difficult in the field.

### In-field processing

An important (short) task was to rename all measurement files, which are automatically stored in folders with the date/time as name, to the specific configuration the measurement was taken in: HH_RR, HV_RU, VV_UU, or VH_UR. We had written down all the measurement start-times and configurations in the field ( Figure 8d). Preliminary in-field processing was performed using MATLAB scripts provided by F. Pattyn (FROID project) and C. Martin (British Antarctic Survey). Two types of plots were made: a time domain plot to check if the range of the signal is within bounds of 0–2.5 Volt, and a power spectrum to check if a bedrock reflection is found ( Figure 9).

## GNSS measurements and ablation stakes

### Objective

Measure (a) the height and (b) position of ablation stakes to derive local ablation and horizontal velocity. These measurements need to be repeated in the next field mission. We performed two types of GNSS measurements to determine the position of the ablation stakes: (1) high-precision measurements at the ablation stakes at the ApRES locations, obtained with a Trimble NetR9 receiver on a tripod measuring for ~2 hours to achieve sub-centimeter accuracy, and (2) measurement of 69 randomly distributed ablation stakes, with a Trimble ^®^ GeoXH ^®^ 6000, measuring for 5–6 minutes (trade-off between accuracy and speed of measurement; Figure 1). For both modes of GNSS measurements, a reference base station (another Trimble NetR9) was placed over a marker that we installed in a rock on the Yetnuten nunatak next to the camp, measuring ~6–8 hours during a ‘GPS day’ to improve accuracy (differential GNSS).

### Height measurement of ablation stakes

**Figure 10.**
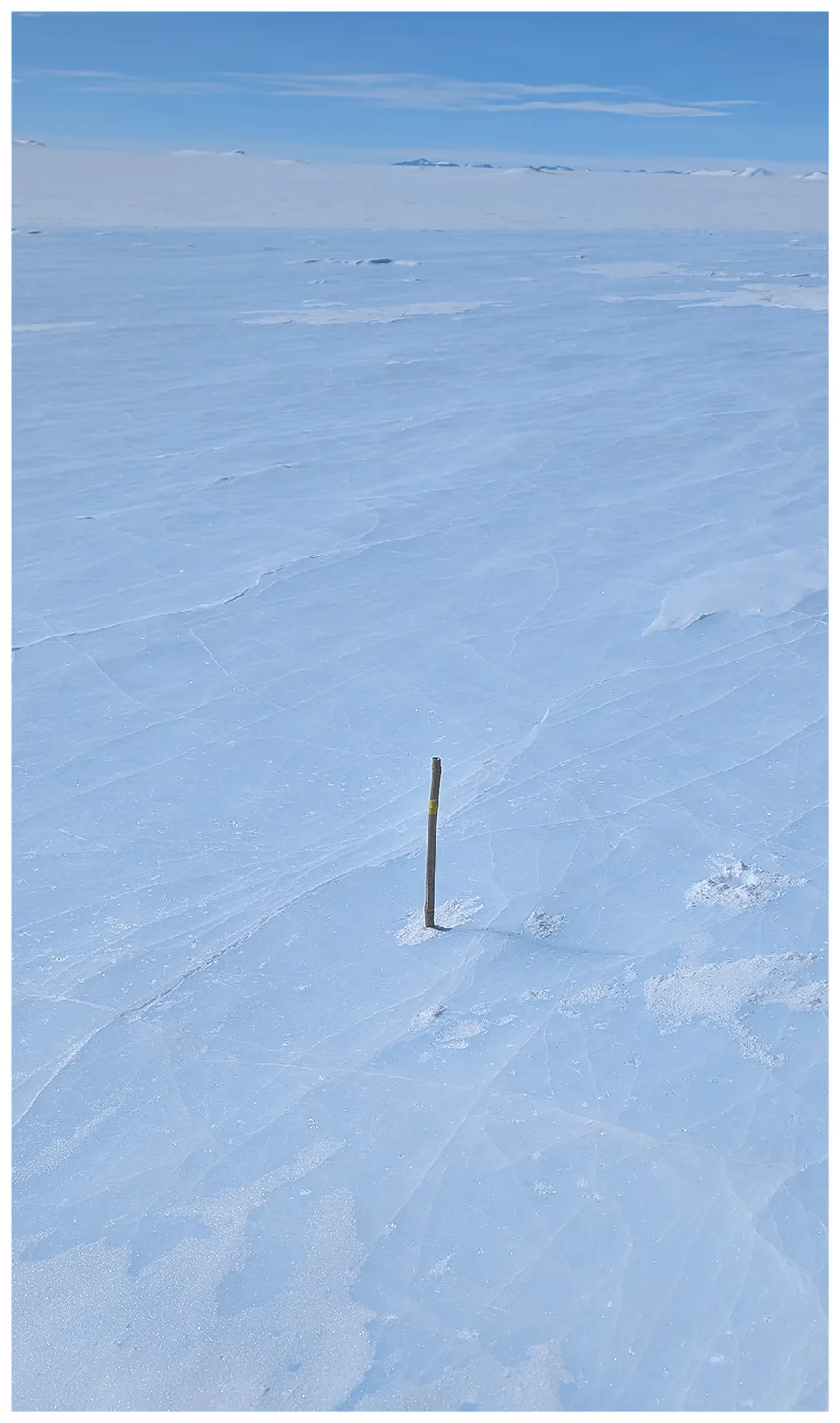
Ablation stake drilled into ice, approximately 20 cm above the ice surface.

Holes of around 1 meter depth were drilled into the ice with an ice auger drilled, after which bamboo stakes of approximately 1.20 m were inserted in the holes, leaving ~20 cm of the stake above the surface ( Figure 10). The height above the ice surface was measured exactly using a standard Stanley tape measure, from the surface of the ice (removing any snow) to the top of the bamboo stake. Repeat measurements of the height above the surface during the next mission will provide ablation rates. Since blue ice areas have a negative surface mass balance, the stake will not become buried. We expect ablation rates in the blue ice areas in this region to be less than 20 cm/year. ^21^ As the second FROID mission is planned to take place two years after the first one, this depth should be sufficient for the stake to remain in the ice (“standing”) upon return. The bamboo stakes were stabilized in the drillhole by re-filling the hole with ice chips and/or snow.

### (1) Trimble GeoXH ^®^ high-precision GNSS field set-up and protocol

**Figure 11.**
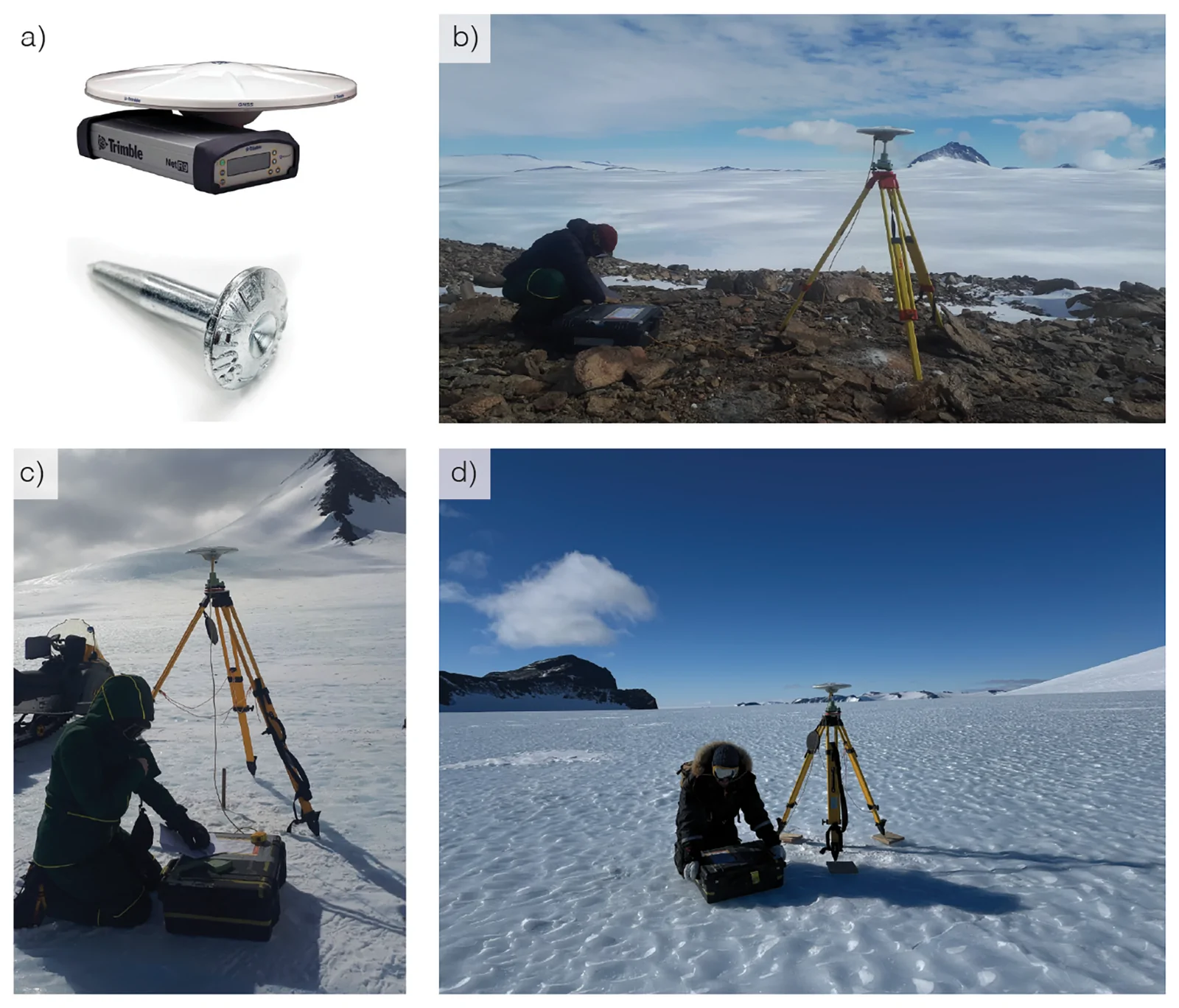
GNSS measurements using (a) Trimble ^®^ NetR9 receiver (grey box) and Zephyr 3 Geodetic Antenna (white disk). (b) Base station: set up on over survey point (shown in (a)) drilled into rock, tripod centered above it. Antenna oriented South. Tripod stabilized with rocks as it was on a ridge with strong (constant) winds. (c) and (d) Rover station: tripod centered above hole of ablation stake. Antenna oriented South; (d) use of wooden base plates for tripod legs to prevent the latter melting into the ice and ease centering.

The base station was set up at a stable location (i.e. large rock) on the nunatak ( Figure 11), away from large metallic objects and with a broad, unobstructed sky view. We installed a survey point nail (in the rock) and set up a tripod centered over the nail.

On each day we performed GNSS measurements in the field, one Trimble receiver was set up at the base station for the whole day (~6–8 hours). Having these longer base station measurements improves accuracy of the ‘rover’ station during postprocessing. The receiver was retrieved at the end of the day to recharge and reheat the battery, and to prevent any potential damage to the antenna due to forecasted strong winds at night.

Installing the Trimble NetR9 receiver:

• Set up tripod centered over the hole of the ablation stake (not over the stake, as it might move) or above the survey point nail. Use three wooden plates with spikes as base for each leg of the tripod on ice.
• Mount antenna, orientation to the South, level position.
• Measure antenna height until stripe on antenna.
• Attach battery and control unit, make sure it is turned on
• Fill log sheet and take photo of setup
• Record for 2 hours

### (2) Trimble ^®^ 6000 GNSS field protocol

**Figure 12.**
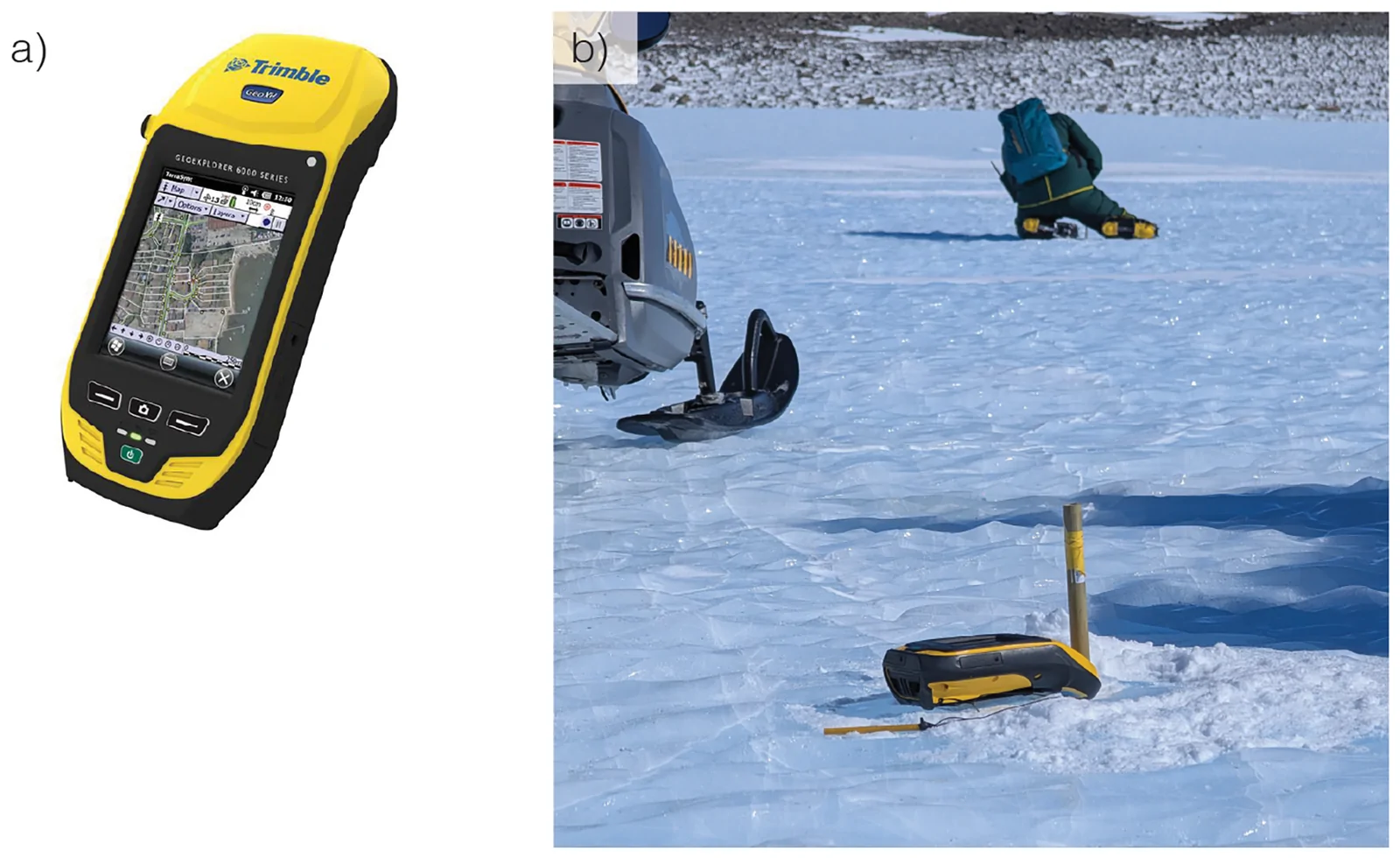
(a) Trimble ^®^ 6000 (b) measuring position of ablation stake. In the back, one team member is taking an ice sample during the waiting time.

This device was used for measuring the position of the distributed ablation stakes ( Figure 12). As these were much higher in number (69) we needed to measure for a shorter duration. Even with the base station to increase accuracy, these measurements might have an uncertainty in the same order of magnitude as the (very small) ice flow speed.

Ablation stake positions were placed in ice patches 2, 3, 4, 6, 8, 9, and measured similarly as the ApRES stakes. The Trimble device was positioned against the stake and measured for at least 5 minutes. In the meantime, three ice samples were taken at ~20 m distance from each other around the stake (following same method as described in section ‘Surface isotope samples’).

### Encountered challenges

**Battery life**. The NetR9 receiver has an internal battery that according to documentation has ‘up to 15 hours of operation’ (at room temperature), but it can also receive an external power source. We always connected a snowmobile-battery to the system (Pb, 12 V/18 Ah). Nevertheless, sometimes the receiver stopped working after a few hours – suggesting it had depended solely on the internal battery, with greatly reduced operational time (2–4 hours) in the cold environment. After troubleshooting we inferred that two issues may have affected our setup. Firstly, the provided external power must be above 9.5 V to provide power; it should be above 12 V for the internal battery to charge. One of our snowmobile batteries did not charge correctly and only reached 8 V, therefore not working as expected.

Secondly, an error message “ *Charger Disabled, Temp Limited*” showed on the Trimble display if the temperature of internal battery got too low (which must remain above 5 °C to charge). This message occurred regularly, even though the receiver and all batteries were stored at room temperature before field deployment. Handwarmers were placed in the box on the receiver to prevent extreme cooling. If the external battery was working properly, measurement could continue without issue. The battery life of the handheld Trimble ^®^ 6000 was about ~3 hours in the field.

**Level position of tripod for NetR9 receiver.** The metal legs of the tripod can cause localized melting and slowly sink into the ice, causing the system to lose its levelled position. This displacement of the tripod was known to be significant on snow/firn surfaces from experience by one of the team members. The effect was not very pronounced on the blue ice surface, but due to the required centimeter-scale precision of these GPS measurements, even small shifts were undesirable. To account for this potential movement, we used three wooden plates with spikes (screws) that could be anchored in the ice (simply by stepping on them), on which the tripod was positioned – changing both the interactive material and the pressure surface area and preventing displacement. Positioning the tripod on wood was also easier than on ice, as it was easier to apply small adjustments for centering and levelling the system.

**Wind conditions.** The high-precision measurements with the Trimble NetR9 receiver were constrained by wind conditions, as strong winds risked destabilizing and potentially damaging the equipment. Therefore, the receiver and antenna of the base station were always retrieved at the end of a day and not set up permanently for the whole expedition (in addition to the battery needing to be charged). The tripod of the base station was left standing for ease of setup. After a night of ~4 bft wind speeds the tripod was blown over.

### In-field processing

Apart from checking if the measurement was successful, no processing was performed in the field (nor at the basecamp). The team conducted the validity check at the basecamp by connecting the Trimble NetR9 receiver to a *Windows* laptop (only supported OS),using the specific IP address to check and retrieve files. The measurement was considered successful if the file was of reasonable size, and there were no other issues or objections encountered during the measurement (such as empty battery issues, or movement of the tripod).

## Meteorite-related activities

Meteorites have been found in the Nils Larsen area during the BELgian Antarctic Research Expedition (BELARE) meteorite reconnaissance expedition in 2022–2023. ^14^ Consequently, we spent a short amount of our time to (a) place and monitor ‘fake meteorites’, (b) to search for actual meteorites, and (c) to fly with an uncrewed aerial vehicle (UAV) to map the blue ice area near the moraine and near PEA. These activities were not the priority of this field campaign, but due to numerous good weather days there was sufficient time to perform them opportunistically.

### Objective

**Figure 13.**
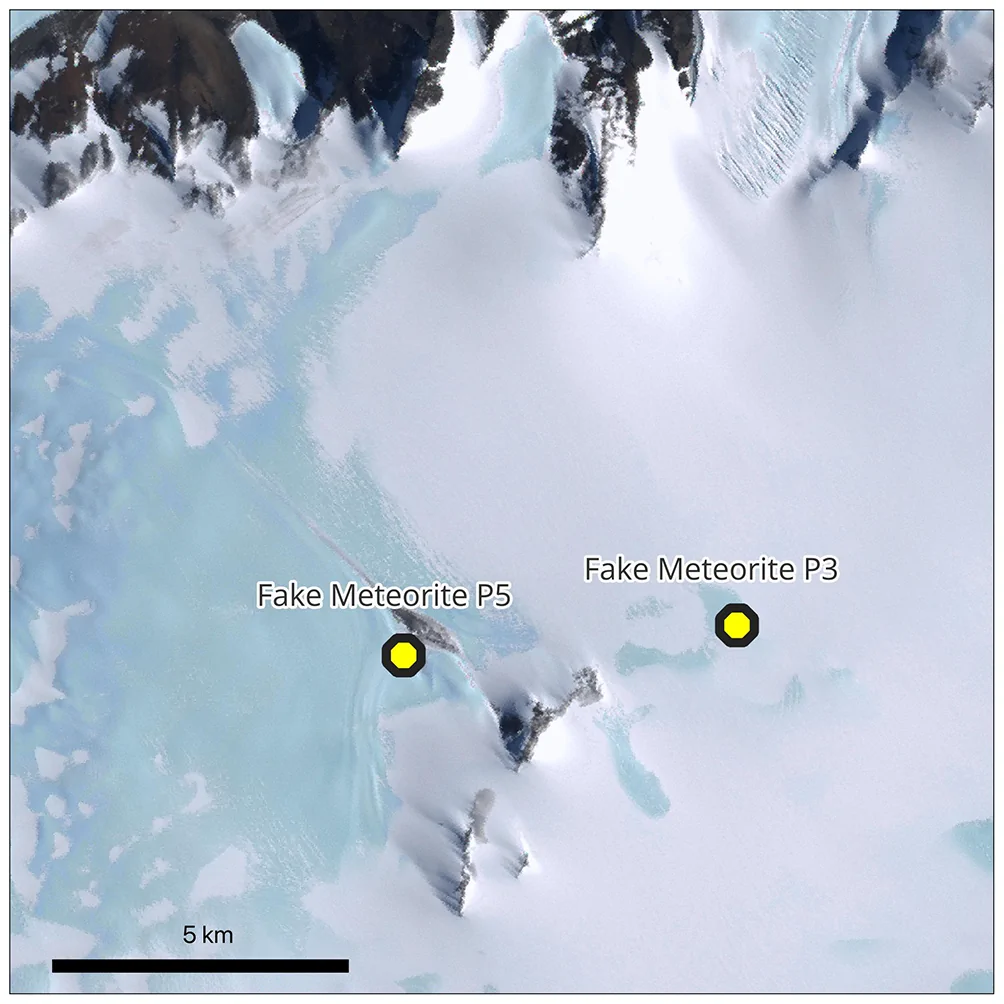
Placement of Fake Meteorites in Patch 5 (P5) and Patch 3 (P3).

(a) To monitor the submergence rate of (iron-rich) meteorites in blue ice, ^22^^,^^23^ we placed two metal cylinders (‘fake meteorites’) of 1.32 kg (± 0.01 kg; 1 SD) at different locations (patch 5 and patch 3; Figure 13) and revisited them regularly to observe how fast they would potentially sink in the ice. This weight is way above the threshold of 200 g to avoid the metal piece being blown away by the wind. ^24^
(b) To find and retrieve meteorites.
(c) To perform initial tests for the use of UAVs to obtain a high-precision orthomosaic, that can be utilized in future research aiming to develop automated searching methods (e.g. via deep learning) to detect meteorites.

### Protocol for ‘fake meteorites’

**Figure 14.**
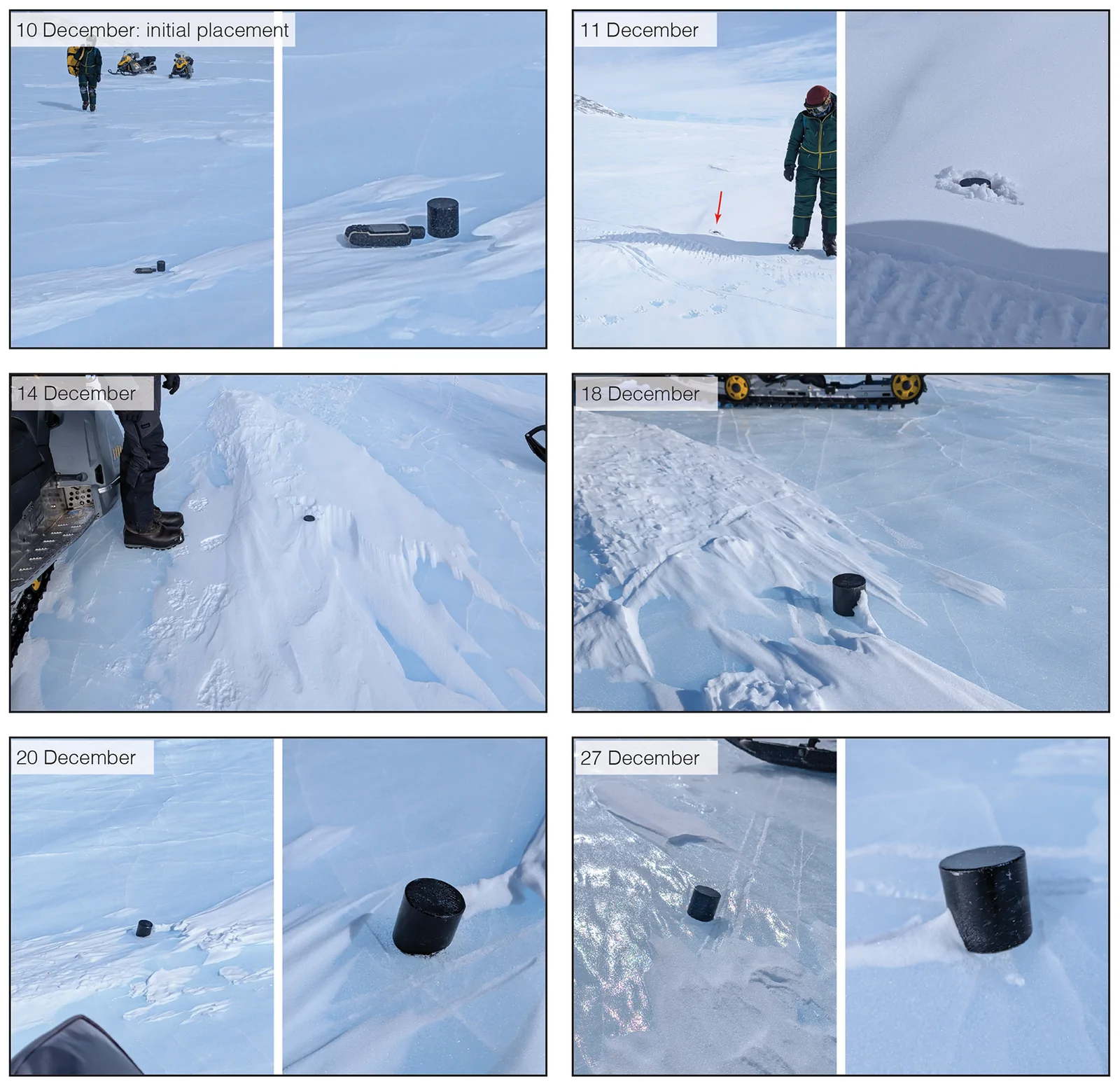
Chronology of observations of Fake Meteorite at Patch 3. Photos of placement and revisits, showing variable snow cover and little submergence.

**Figure 15.**
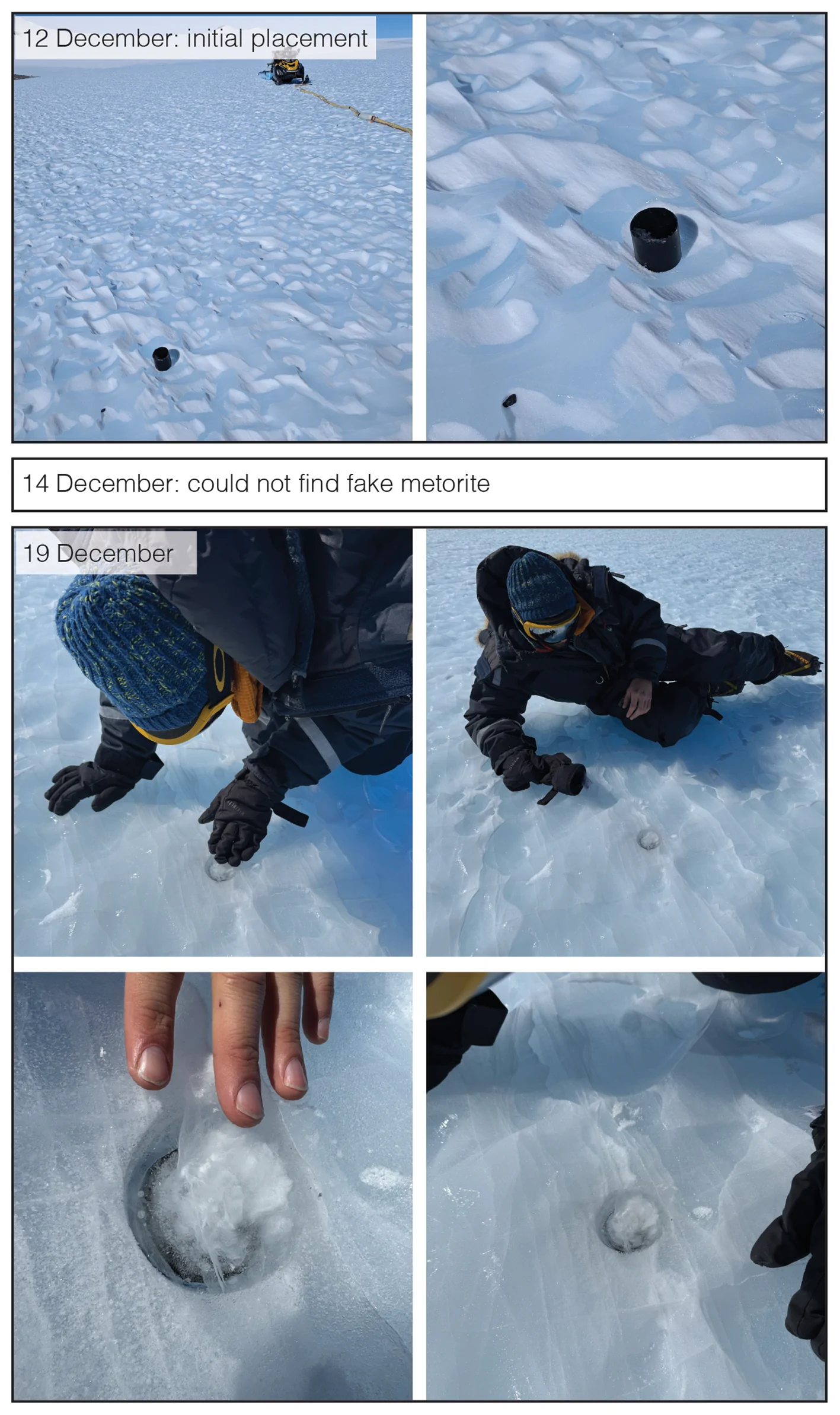
Chronology of observations of Fake Meteorite at Patch 5. Photos of placement and revisit; found the fake meteorite fully submerged on the 19th of December with 2–3 cm (refrozen) ice on top.

Two metal cylinders painted in black were used to represent iron-rich meteorites, referred to as ‘fake meteorites’. These cylinders were placed at the ice surface at the start of our campaign and revisited regularly to observe the height above the surface ( Figure 13). The fake meteorites remain in the field and will be revisited during the next FROID field campaign. The fake meteorites in Patch 3 stayed at the surface throughout this expedition, with dynamic snow cover ( Figure 14); the fake meteorite in Patch 5 had submerged completely into the ice when revisited on 19 December ( Figure 15). It sank presumably on the warm event of 14 December 2024 when temperatures were around −8 to −6 °C and liquid water was observed at the surface of the ice. On this day, two team members had already attempted to revisit the fake meteorite but could not find it.

### Protocol for meteorite search

We spent 6 hours in total (3 × 2 hour) in a dedicated meteorite search in the moraine close to our camp. At other times, during all our activities, we were on the look-out to spot potential meteorites, during snowmobile travel and along the isotope sampling transects. We brought potential samples back to camp to measure with a MetMet, designed to measure the magnetic susceptibility and used as an indication in the field to determine if a sample could be a meteorite or not, as none of the field members is a meteorite expert. ^25^ None of the samples turned out to be meteorites following verification by the ULTIMO meteorite team that was visiting the Belgica Mountains in a parallel field mission.

The collection of each potential meteorite went as follows: (1) Take pictures of sample before disturbing it, (2) write sample ID on ice (initials of finder + date YYMMDD + sample number, e.g. MI24121801) and take a new picture including sample and ID, (3) mark location on GPS, (4) scoop sample into a plastic bag without touching it in any way (apart from the inside of the bag). Shake any loose snow out of the bag. (5) Store in cold area, keep sample frozen. ^13^ Magnetic susceptibility measurements were made later, keeping the sample in the bag.

In our dedicated search, we walked along the moraine in pairs of two, no more than two hours at a time. We walked at the edge of the moraine, working on the assumption that meteorites blown by the wind would be deposited near the edge, as well as that within the moraine the meteorites are even harder to spot between abundant terrestrial rocks, and providing a logistically convenient approach to walk back and forth along different paths.

### Protocol for UAV flights

**Figure 16.**
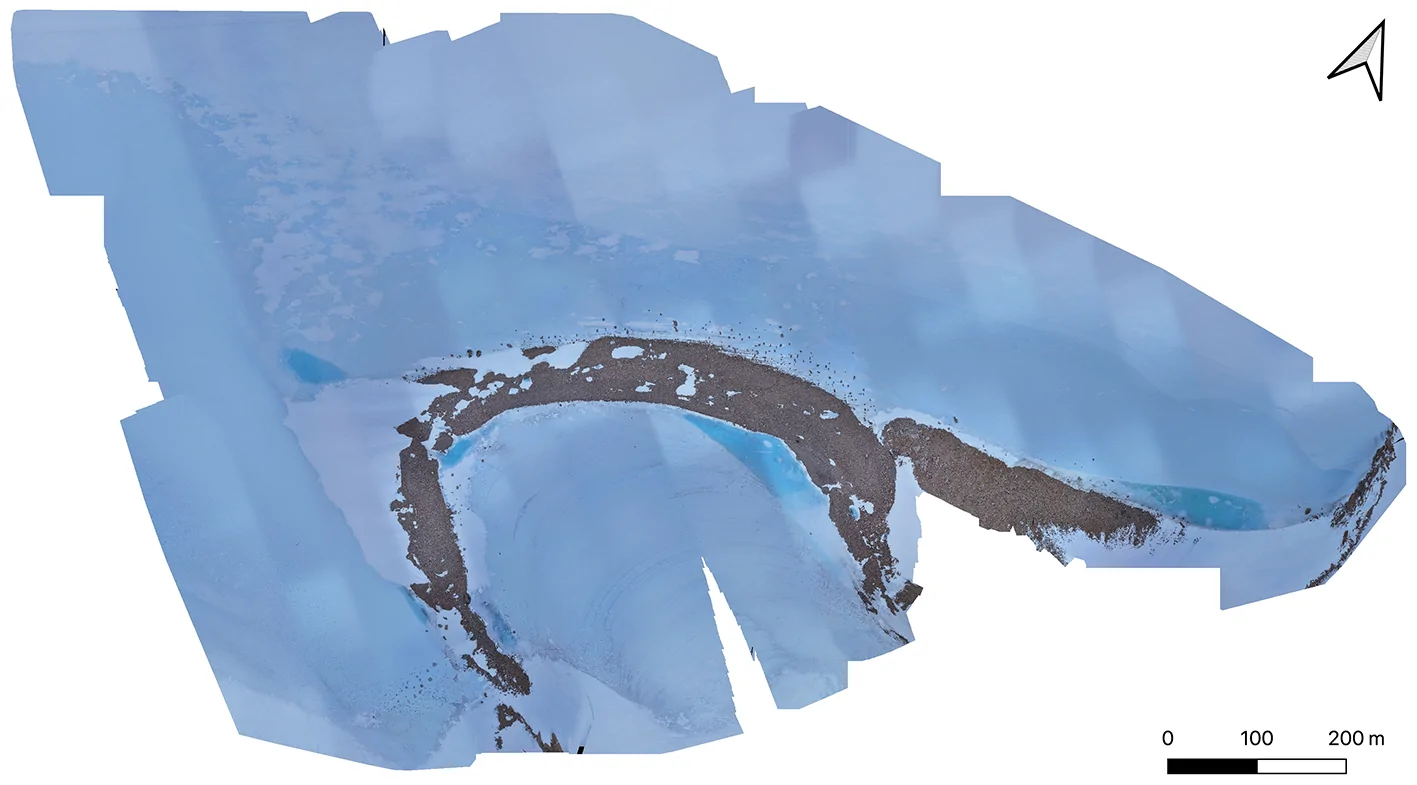
Orthomosaic of DJI Air 3 UAV images near Princess Elisabeth Antarctica, created with Pix4D software.

The UAV, a DJI Air 3, was used to fly a small region over the blue ice area of patch 4 and 6 as well as the edges of the moraine. **Settings**: flight speed of 5 m/s, height of 30 m, flying a grid of approximately 20 m distance, photography every 2 seconds. Aiming for an image overlap of roughly 80% (required for constructing orthomosaic) and a spatial resolution of 1 cm in the images. ^26^ The flights over patches 4 and 6 allowed us to test the limits of the software and the UAV in these harsh field conditions, and this informed a subsequent flight at the PEA station over the blue ice area West of Utsteinen nunatak in wich we successfully mapped an area of approximately 4 km ^2^ in 5 flights (~20 minute per flight; Figure 16). We then created an orthomosaic from the images with the Pix4D software. ^27^

### Encountered challenges

**Figure 17.**
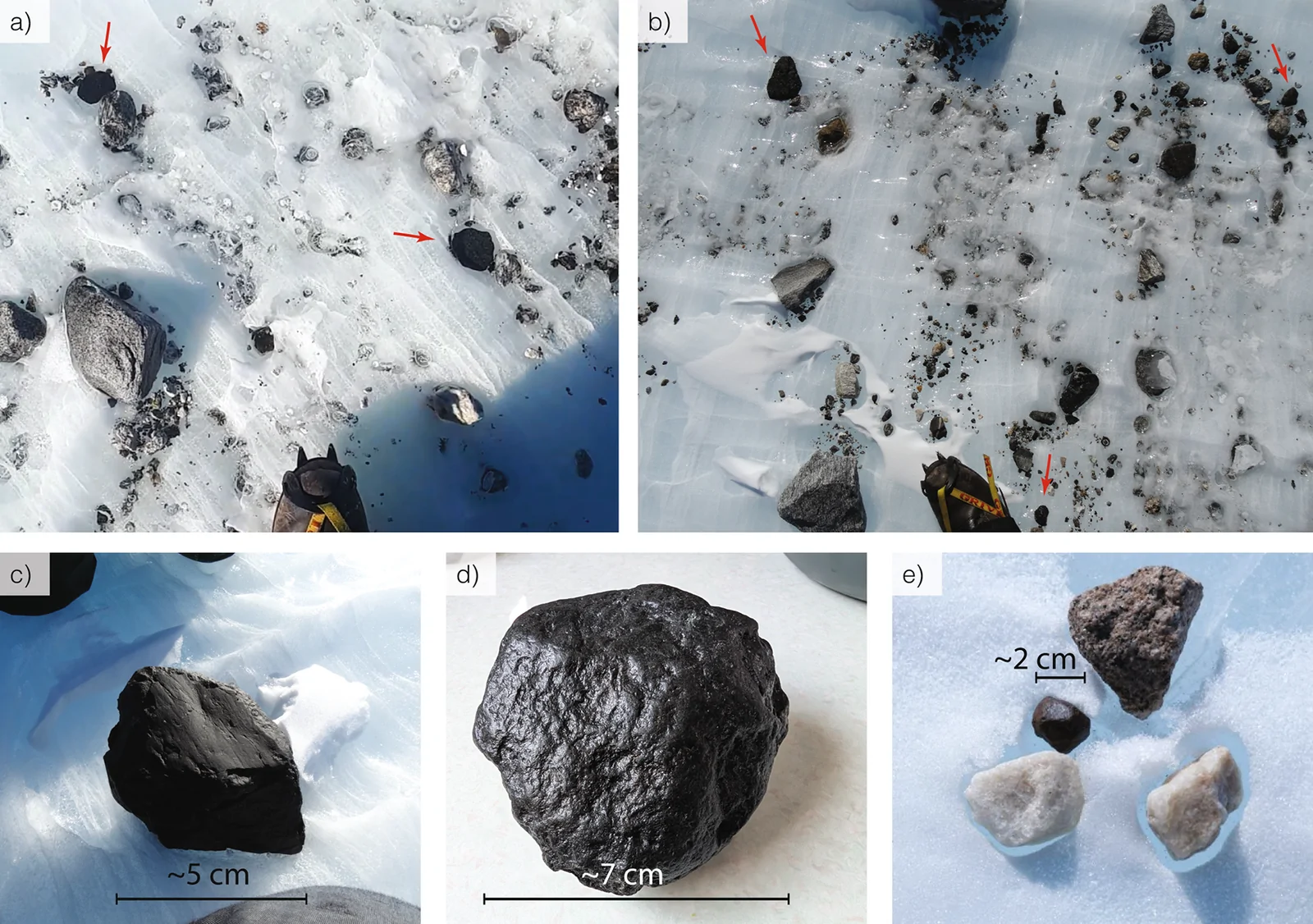
Field pictures from meteorite search in the moraine. (a) and (b) show examples of samples we identified as potential meteorites in their surroundings, (c) and (d) show close up examples of potential meteorites. All examples (a–d) were not actual meteorites. (e) shows an example of a confirmed meteorite found by the ULTIMO team in the nearby Belgica Mountains (the center black item, surrounded by three rock samples (white/gray/brown), as reference.

The geology of the area exposes black amphibolite rocks at the surface, which are difficult to distinguish from meteorites at first glance. ^28^ This made searching the moraine extra complicated with many false positives ( Figure 17). Out on the blue ice, we did not encounter exposed meteorites. Two years prior, a meteorite expedition was conducted in this area and collected three meteorites. ^14^ A hypothesis is that the unique specimens on debris-free ice (i.e., ‘easy to spot’) are already collected. Alternatively, this could show that ‘opportunistic’ meteorite spotting is not effective, and a thorough search approach with experienced specialists is required to successfully find meteorites on large blue ice areas. Lastly, just as the patch 5 ‘fake’ meteorite sank into the ice, meteorites in the area may also have been submerged, making them virtually impossible to detect. During warm events, we observed many terrestrial rocks sinking partially or fully into the ice.

The UAV flights were limited by wind strength, which should remain below 3 bft to safely fly with the deployed UAV (DJI Air 3). The cold was not as limiting a factor, and batteries were always stored in the warm living container.

## Conclusion

This report documents the first FROID field campaign in the Nils Larsen blue ice area and presents the successful acquisition of a comprehensive set of observations aimed at identifying suitable sites for shallow drilling of old ice near Princess Elisabeth Station. Through a combination of ice coring, radar surveys, GNSS measurements and surface isotope sampling, we obtained the first set of observations to construct a detailed overview of the investigated blue ice patches. We also highlighted several methodological and logistical challenges associated with operating scientific equipment in harsh Antarctic blue ice environments, including wind limitations, equipment degradation, calibration issues, and difficulties related to drilling fractured blue ice. These experiences will be used in planning our next mission and may be of use for other scientists planning their next field season. The collected datasets and analysis of ice samples will form the basis for selecting a drilling site for the follow-up campaign and will contribute to a broader understanding of blue ice dynamics and their potential preservation of old ice in East Antarctica.

## Ethics and consent

All field activities, processing and publishing of results has been and will be done in accordance to the Antarctic Treaty, and in particular the Madrid Protocol to the Treaty (1998). Consent was not required.

## Consent to publish

Everyone featured in the video has given consent to publish the material.

## Data and software availability

### Source data

In the planning phase of this fieldwork, we made use of existing datasets that are publicly available: ice flow velocity from MEaSUREs campaign ^6^ ( https://doi.org/10.5067/PZ3NJ5RXRH10), ice thickness data from Bedmachine ^4^ ( https://doi.org/10.5067/E1QL9HFQ7A8M) and Bedmap2 ^5^ ( https://www.bas.ac.uk/project/bedmap-2/#data), Blue Ice Area outlines from 29 and 30.

### Underlying data

Zenodo. ‘Data for “Initial survey of blue ice areas for the recovery of old ice in the Sør Rondane mountains (Dronning Maud Land, East Antarctica)”’: https://doi.org/10.5281/zenodo.17542193. ^31^

This project contains the following underlying data:

- *Survey_plans.zip:* contains.gpx and.shp files with the planned field tracks or points, as shown in Figure 1).
- *Surveyed_points.*zip: contains.gpx and.shp files with the logged (handheld) waypoints (e.g. for ice isotope samples, Figure 8 or fake meteorite placement, Figure 14) and all the skidoo tracks by the four field scientists.
- *Overview_planned.*pdf and *Overview_surved.png* shows the map of the points in both zip folders.

Data are available under the terms of the Creative Commons Attribution 4.0 International license (CC-BY 4.0).

The collected physical ice cores and water isotope samples in this fieldwork will be analyzed in labs in Belgium, France and China, and all resulting data will then be published in open access repositories attached to respective manuscripts, in accordance with the Antarctic Treaty. The ice samples will be stored at the Glaciology lab of Université Libre Bruxelles after analyses is completed.

### Extended data

Zenodo. ‘Data for “Initial survey of blue ice areas for the recovery of old ice in the Sør Rondane mountains (Dronning Maud Land, East Antarctica)” https://doi.org/10.5281/zenodo.17257665 ^31^

This project contains the following underlying data:

• A video montage of work tasks in the field.
• Templates for ApRES measurements.
• GPS measurements, and.
• The table with ice core temperatures (Table 1)

Data are available under the terms of the Creative Commons Attribution 4.0 International license (CC-BY 4.0).

Analysis code for GPR processing steps is available from https://github.com/VeronicaTollenaar/GPR_processing and archived at https://doi.org/10.5281/zenodo.17534240 (GPR, CC-BY 4.0 license). ^32^

## Software availability

Source code with code relevant for the UAV flights is available at https://github.com/VeronicaTollenaar/UAV_grid and archived at https://doi.org/10.5281/zenodo.17534234 (UAV, MIT license). ^26^
