# Supplementary material for: Initial survey of blue ice areas for the recovery of old ice in the Sør Rondane mountains (Dronning Maud Land, East Antarctica)
Source: Open Res Eur. 2026 Jul 23;6:17. Originally published 2026 Jan 9. [Version 2] doi: 10.12688/openreseurope.21562.2 (PMC13555041; doi:10.12688/openreseurope.21562.2)
Supplement: Peer review history

# Reviewer report: Reviewer response for version 2

**DOI:** 10.21956/openreseurope.26172.r77914

**Published:** 2026-08-06

**Authors:** Kate Winter

**Affiliations:** 1 Northumbria University, Newcastle upon Tyne, England, UK

Dear authors,

 Thank you for taking the time to address all reviewer comments. 

 The manuscript is now suitable for indexing.

 I enjoyed watching the video montage in the supplementary data link. A nice addition - and a useful resource for others who may wish to conduct similar work in the future. 

 Kind regards,

 Dr Kate Winter

Is the case presented with sufficient detail to be useful for teaching or other practitioners?

Yes

Is the work clearly and accurately presented and does it cite the current literature?

Yes

If applicable, is the statistical analysis and its interpretation appropriate?

Not applicable

Are all the source data underlying the results available to ensure full reproducibility?

Partly

Are the conclusions drawn adequately supported by the results?

Partly

Is the background of the case’s history and progression described in sufficient detail?

Yes

Reviewer Expertise:

Antarctic fieldwork, Blue Ice Area research, GPR, ApRES, Ablation Stake measurements, Drone mapping

I confirm that I have read this submission and believe that I have an appropriate level of expertise to confirm that it is of an acceptable scientific standard.

# Reviewer report: Reviewer response for version 2

**DOI:** 10.21956/openreseurope.26172.r77915

**Published:** 2026-07-25

**Authors:** Julien A. Bodart

**Affiliations:** 1 University of Bern, Bern, Switzerland

Dear Editorial team and Authors,

 I am satisfied with the responses of the authors to my comments. I recommend this paper to be accepted as is.

 Many thanks for engaging with all aspects of the reviewing process. 

 Best wishes,

 Dr Julien Bodart

Is the case presented with sufficient detail to be useful for teaching or other practitioners?

Yes

Is the work clearly and accurately presented and does it cite the current literature?

Yes

If applicable, is the statistical analysis and its interpretation appropriate?

Not applicable

Are all the source data underlying the results available to ensure full reproducibility?

Yes

Are the conclusions drawn adequately supported by the results?

Partly

Is the background of the case’s history and progression described in sufficient detail?

Yes

Reviewer Expertise:

Radar, Field glaciology, Climate, Ice-sheet modelling, Satellite data analysis

I confirm that I have read this submission and believe that I have an appropriate level of expertise to confirm that it is of an acceptable scientific standard.

# Reviewer report: Reviewer response for version 1

**DOI:** 10.21956/openreseurope.23325.r71005

**Published:** 2026-04-02

**Authors:** Kate Winter

**Affiliations:** 1 Northumbria University, Newcastle upon Tyne, England, UK

This is more of a field report than a traditional scientific paper analysing data and placing findings into a wider context, but it is still very useful to the cryospheric community as it details how to live and work in Antarctica - performing a variety of research tasks which are commonly undertaken in cryospheric research (but rarely described in this much detail). This report will be useful to those wishing to conduct similar work, possibly with less experienced field scientists. 

 The paper is generally quite well written, with supportive figures. I did find the weather limitation sections quite repetitive, so I wonder if it's best to replace these headings with 'research limitations' where you can talk more broadly about your limitations e.g. through field kit choices (batteries?) as well as the weather (which needn't be so repeated). The figures require more locational information and scale. Some of this can be added in the caption.

 The manuscript ends very abruptly with no conclusions. I suggest you write a summary paragraph to conclude your findings and briefly outline your proposed next steps.

 Some minor suggested changes:

 In the abstract: What does QUOI stand for?

 In the plain language summary (end of paragraph 1): you should be more climate specific and say something like “But the climate record of most blue ice areas in Antarctica have yet to be extracted” (as many blue ice areas have been studied from satellites/for meteorites, even with radar etc).

 Introduction: After cul-de-sac change “and” to “where” surface velocity “is”.

 Full stop required at the end of meteorite collection.

 Overview: the team, the area and logistics

 Provide a lat/long for Princess Elisabeth Antarctica. Mention what your field camp looked like, as the container camping approach you used must have supported battery charging (with a generator or solar panels?), GPR processing on a laptop and your ability to keep equipment warm (compared to more traditional camping). A photo would be nice too. 

 Figure 2: MALA GPR settings would be easier to view in a table.

 Figure 3 (and associated text): It would be useful to highlight where the GPS is located on your radar setup and what the reported accuracies are in X,Y and Z. 

 In-field processing: In-field processing was completed at the basecamp (rather than done). Check this throughout. The word "done" is overused in the text and not very appropriate. 

 Figure 4: Caption should reference the red line and what is shows (a manually picked ice/bed interface?). Picked in ReflexW or some other software?

 You mention QGIS when you discuss the radar transects but it's unclear what you use it for. Presumably just for plotting up the location(s) of the radar lines from the GPS?

 In(Visible) bedrock reflections: Write "This was conducted in patch 5"… (rather than “done”)

 Figure 5 caption: Note the approximate size of the hammer in figure 5a for scale reference. Though it would be better to take more photos with a clear scale in the field (for next season!).

 Field execution (ice cores) or elsewhere:

 Please mention how you measured ice temperature. It seems like you measured the temperature in between ice core sections rather than the borehole which seems like an unusual approach to me.

 What equipment did you use for the drilling? Is it something someone could buy ‘off-the-shelf’ from a company, or does it need to be constructed in-house?

 The term pancake ice usually refers to sea ice. I’d reword this, maybe to “lenses of ice”.

 Challenges with equipment (coring)

 What do you propose to use instead of a stapler if you were doing this field research again? I would try to bring these types of suggestions into all your limitations sections e.g. how would you reduce limitations in the future?

 Water sampling: Was the ice screw cleaned in between samples? And if so, with what? What were the ice screws made of? Could they contaminate the melted ice in any way? Will you account for this in your analysis?

 Figure 8: Caption should say which patch this sampling was conducted at (with reference to Figure 1).

 When you mention sleeping containers (below figure 8) you should give more detail about what these are – e.g. modified, insulated shipping containers on skis? See earlier comment.

 Figure 9: Why was the laptop optional? Is it not critical for setting off and then checking the ApRES? I see you mention this later on... I would therefore suggest you bring a Toughbook to the field (it’s a laptop that works well in the cold).

 Figure 14: Provide patch name in the caption (or latitude and longitude for each fake meteorite).

 Fake meteorite suggestion – in the future, could you place a time lapse camera on the fake meteorites to more accurately record how quickly they melt in?

 Fake meteorites – it is unclear how you plan to find the fake meterorites in the future and how you will analyse any displacement if you do find them.

 Figure 18 – can you give an approximate scale for each photo? Unclear if captioning is correct as e seems to refer to one meteorite, when there are four rocks in panel e.

Is the case presented with sufficient detail to be useful for teaching or other practitioners?

Yes

Is the work clearly and accurately presented and does it cite the current literature?

Yes

If applicable, is the statistical analysis and its interpretation appropriate?

Not applicable

Are all the source data underlying the results available to ensure full reproducibility?

Partly

Are the conclusions drawn adequately supported by the results?

Partly

Is the background of the case’s history and progression described in sufficient detail?

Yes

Reviewer Expertise:

Antarctic fieldwork, Blue Ice Area research, GPR, ApRES, Ablation Stake measurements, Drone mapping

I confirm that I have read this submission and believe that I have an appropriate level of expertise to confirm that it is of an acceptable scientific standard, however I have significant reservations, as outlined above.

# Response

**Published:** 2026-05-29

**Authors:** Maaike Izeboud

**Affiliations:** Water and Climate, VUB Faculty of Engineering, Brussels, Brussels, Belgium

This is more of a field report than a traditional scientific paper analysing data and placing findings into a wider context, but it is still very useful to the cryospheric community as it details how to live and work in Antarctica - performing a variety of research tasks which are commonly undertaken in cryospheric research (but rarely described in this much detail). This report will be useful to those wishing to conduct similar work, possibly with less experienced field scientists. 

 We thank the reviewer for their effort and are happy to hear they think it is useful to other field scientists, as that was our intent.

**[R3-1]** The paper is generally quite well written, with supportive figures. I did find the weather limitation sections quite repetitive, so I wonder if it's best to replace these headings with 'research limitations' where you can talk more broadly about your limitations e.g. through field kit choices (batteries?) as well as the weather (which needn't be so repeated). The figures require more locational information and scale. Some of this can be added in the caption.

- We concur that the weather limitations section is largely the same in each section, and appreciate the suggestion of the reviewer. We have implemented this by (a) incorporating a short description on overall ‘weather limitations’ (such as hypothermia risk) into the Overview section “General approach in the field” and incorporated equipment-specific weather limitations into the existing ‘encountered challenges’ section, when relevant.
- We have included a coordinate frame to Figure 1 and added a size indication to Figure 18.

**[R3-2]** The manuscript ends very abruptly with no conclusions. I suggest you write a summary paragraph to conclude your findings and briefly outline your proposed next steps.

**Some minor suggested changes:**

**[R3-3]** In the abstract: What does QUOI stand for?

 Rather than detailing ‘QUOI’ in the abstract, we have removed this part of the sentence from the abstract (‘funded by the Belgian Science Policy Office (BELSPO), with logistical support from the International Polar Foundation (IPF) and additional funding from the QUOI project’). The QUOI project and acronym is introduced fully in the Introduction: Quest for world’s oldest ice.

**[R3-4]** In the plain language summary (end of paragraph 1): you should be more climate specific and say something like “But the climate record of most blue ice areas in Antarctica have yet to be extracted” (as many blue ice areas have been studied from satellites/for meteorites, even with radar etc).

 Agreed, this has been implemented.

**[R3-5]** Introduction: After cul-de-sac change “and” to “where” surface velocity “is”.

 Full stop required at the end of meteorite collection.

 Implemented.

**[R3-6]** Overview: the team, the area and logistics. Provide a lat/long for Princess Elisabeth Antarctica. Mention what your field camp looked like, as the container camping approach you used must have supported battery charging (with a generator or solar panels?), GPR processing on a laptop and your ability to keep equipment warm (compared to more traditional camping). A photo would be nice too.

 We have added this description of the camp: “ *The basecamp consisted of two modified and insulated shipping containers for living/sleeping supported with solar panels and heating system, one container to store scientific equipment and one reefer container for the ice core samples”*. We don’t think a photo is necessary and that we have enough figures as it is, so have not included it in the main file but we include a photo here for the reviewer:   

**[R3-7]** Figure 2: MALA GPR settings would be easier to view in a table.

 Agreed, it has been converted into a table (Table 1).

**[R3-8]** Figure 3 (and associated text): It would be useful to highlight where the GPS is located on your radar setup and what the reported accuracies are in X,Y and Z. 

 We have added in the text “ *The MALA system includes a connected* *GPS receiver (GLOBALSAT BU-353S4) which immediately co-registers coordinates (latitude, longitude) and surface elevation to each datapoint (accuracy up to 5 m)” *and have updated Figure 3a to include it in the schematic.

**[R3-9]** In-field processing: In-field processing was completed at the basecamp (rather than done). Check this throughout. The word "done" is overused in the text and not very appropriate. 

 Thanks for pointing this out. We have revised use of ‘do/done’ throughout the text. In some cases the verb was replaced with another one (e.g. ‘processing was *completed/performed*’ and ‘measurements were *taken’) *and in some cases the sentences was restructured and the verb omitted.

**[R3-10]** Figure 4: Caption should reference the red line and what is shows (a manually picked ice/bed interface?). Picked in ReflexW or some other software?

 Yes the red line is picked in ReflexW. It has been added to the caption: *“Red line shows preliminary picking of bedrock reflection in ReflexW”*

**[R3-11]** You mention QGIS when you discuss the radar transects but it's unclear what you use it for. Presumably just for plotting up the location(s) of the radar lines from the GPS?

 Indeed it was used for visualizing. We’ve clarified in text: “ *In-field processing was completed at the basecamp with a simple pipeline using the ReflexW software (* *Figure 4* *) (signal processing; picking bedrock reflections), python (simple calculations and data aggregation) and QGIS (visualisation)*”

**[R3-11]** In(Visible) bedrock reflections: Write "This was conducted in patch 5"… (rather than “done”)

 We have restructured the sentence to “ *For longer stretches without any visible bedrock reflection, in patch 5, patch 1 and patch 7, we adjusted* …”

**[R3-12]** Figure 5 caption: Note the approximate size of the hammer in figure 5a for scale reference. Though it would be better to take more photos with a clear scale in the field (for next season!). Point well taken! We have added “The worktable has a width of 1 m” to the caption”. **[R3-13]** Field execution (ice cores) or elsewhere:

- Please mention how you measured ice temperature. It seems like you measured the temperature in between ice core sections rather than the borehole which seems like an unusual approach to me.

We have specified in the field execution section: *“…,  we additionally measured the ice temperature at various depths (* *Table 1* *), by drilling a small hole in the ice core and placing a thermometer in it”*. This was just an approximate measurement to get an idea of ice temp, and lighter than a borehole temperature device.

- What equipment did you use for the drilling? Is it something someone could buy ‘off-the-shelf’ from a company, or does it need to be constructed in-house? 

The barrel was handcrafted by the technical team at the Université libre de Bruxelles. This has been added to the manuscript.

- The term pancake ice usually refers to sea ice. I’d reword this, maybe to “lenses of ice”.

Agreed, we have removed it from the text as it was not a necessary term, or referred to it as ‘narrow disks’.

**[R3-14]** Challenges with equipment (coring): What do you propose to use instead of a stapler if you were doing this field research again? I would try to bring these types of suggestions into all your limitations sections e.g. how would you reduce limitations in the future?

 Good suggestion. In some places this was already provided, but we have checked the manuscript and have added the following advice:

- W.r.t the stapler: “ *We advise to bring a stapler that has easy access to the staple-area and/or can be opened in order to make small repairs”*
- W.r.t recovering a dropped barrel: “ *The barrel dropped both times due to faulty connections of the extension rods, which should always be checked thoroughly before lowering the system, to prevent this from occuring. Connection pins should be kept dry.”*
- W.r.t GPR: “Equipment degradation **: **due to the hard but rough surface the system endured a lot of (vibrational) impact. We had spare equipment of batteries, fibre optic cable and ethernet cables, all of which were used by the end of the campaign. **”**

**[R3-15]** Water sampling: Was the ice screw cleaned in between samples? And if so, with what? What were the ice screws made of? Could they contaminate the melted ice in any way? Will you account for this in your analysis?

 The screws are of stainless steel. The samples are filtered before measuring the oxygen and hydrogen isotopes, so any contamination of the material and/or dust in the ice is removed. Only contamination could be if the screw is not completely empty when taking the sample, but given the size of the sample (~40 g), these contaminations are negligible for detecting a signal that captures temperature differences of ice formed during glacial and interglacial periods.

**[R3-16]** Figure 8: Caption should say which patch this sampling was conducted at (with reference to Figure 1).

 Added; it was Patch 2.

**[R3-17]** When you mention sleeping containers (below figure 8) you should give more detail about what these are – e.g. modified, insulated shipping containers on skis? See earlier comment.

 Indeed; see response to earlier comment **R3-6**.

**[R3-18]** Figure 9: Why was the laptop optional? Is it not critical for setting off and then checking the ApRES? I see you mention this later on... I would therefore suggest you bring a Toughbook to the field (it’s a laptop that works well in the cold).

 The laptop is not required for starting or stopping the ApRES measurements, only when doing calibration.

**[R3-19]** Figure 14: Provide patch name in the caption (or latitude and longitude for each fake meteorite).

 Added! Specified placement in Patch 5 and Patch 3.

**[R3-20]** Fake meteorite suggestion – in the future, could you place a time lapse camera on the fake meteorites to more accurately record how quickly they melt in?

 Yes, that would be very valuable.

**[R3-21]** Fake meteorites – it is unclear how you plan to find the fake meteorites in the future and how you will analyse any displacement if you do find them.

 The region consists of very slow moving ice (~<1 m/yr) so the saved location will help direct us. We do not expect the cilinders to sink deep into the ice, as the assumption is that if they sink, they will sink during a day/period when their localized melt submerges them, but won’t cause them to submerge indefinitely once they are a few cm under the ice (and thus will remain visible).

**[R3-22]** Figure 18 – can you give an approximate scale for each photo? Unclear if captioning is correct as e seems to refer to one meteorite, when there are four rocks in panel e.

 Yes, we have added a size indication. We acknowledge the caption was unclear, and have clarified panel (e) shows the meteorite, which is the center black item, surrounded by three rock samples (white/gray/brown samples)

# Reviewer report: Reviewer response for version 1

**DOI:** 10.21956/openreseurope.23325.r71011

**Published:** 2026-03-27

**Authors:** Julien A. Bodart

**Affiliations:** 1 University of Bern, Bern, Switzerland

Dear Editor and Authors,

 Please find attached my review of Izeboud et al. with manuscript title “Initial survey of blue ice areas for the recovery of old ice in the Sør Rondane mountains (Dronning Maud Land, East Antarctica)”.

 The manuscript is a field report, not a science paper per se. It describes the first of two field seasons in Dronning Maud Land area, aimed at recovering more information on the blue ice areas around the Princess Elizabeth Station. The report describes the key methods and equipment used and discusses the limitations (particularly weather-related) associated with each of the methods. I found it interesting to read as it is relatively rare nowadays to publish field reports which can help and guide future surveys attempting to do the same thing.

 This paper is well written, and the figures are clear for the most part. I only have a few minor comments regarding the text or figures but overall, I would recommend this paper to be indexed in Open Research Europe with minor revision.

 With best wishes,

 Dr. Julien Bodart

 -------------------------------------------------------

**General comments**

- I think the only thing I am missing is a conclusion section, describing the key achievements from the field season, and what has been learned for season 2. Perhaps some limitations associated with the methods or equipment used and which will be taken forward for the next field season?

-------------------------------------------------------

**Line-by-line comments:**

- Abstract: Please state what the abbreviation “QUOI” stands for. Also remove “…” in “CO2, CH4, N2O ...)” everywhere in the paper and either state all or say “amongst others”.

- “These products provided a preliminary understanding of the area but were too coarse and uncertain to capture local processes that are important to determine a drilling site.” – please specify this is only applicable to the use of bedmachine/bedmap2/measures, and not the AWI radar profiles used, as AWI radars have a much higher resolution that is enough to guide ice core drilling.
- “In addition, estimated terrestrial age of meteorites found on the blue ice in the Nils Larsen ...” – please provide a reference for this sentence
- “with four patches considered to have the highest priority (Patch 9, Patch 8, Patch 4 and Patch 2; Figure 1b).” – why? Explain
- Figure 1: it would be useful to provide at least one figure with coordinates on x and y axis.
- “A surface ice sample transect along the direction of ice flow, sometimes two transects.” Since you state that each patch had the same set of measurements at every patch, then maybe say “At least one surface ice sample transect along the direction of ice flow”.
- “‘fake meteorites’” – please explain
- “We used a MALA RTA of 25 MHz with an antenna distance of 6 m to measure ice thickness, from which bed topography can be derived.” – this doesn’t really make sense. Bed topography is imaged, which by subtracting with surface return gives ice thickness. Also, maybe it should be bed elevation, in which case you should state that this is obtained by subtracting ice thickness from surface elevation observed with GNSS at the surface.
- Instead of Figure 2, I would add a table which describes the key parameters used.
- “To identify areas with ice thickness < 200 m and ideally a ‘cul-de-sac’ topography, used to determine the site for next field mission.” This sentence does not make sense grammatically. Also is “cul-the-sac” topography really a well-known thing? Is there an equivalent in English that would be easier for non-French speakers to understand?
- “Moreover, initial processing was done in the basecamp (using ReflexW software14) to select suitable locations for the 10-m depth ice cores in each patch.” I would rephrase this sentence as you mention later what the processing consisted of.
- “In-field processing” for the radar: please also state how you acquired the gps positions of each trace (GNSS station embedded on top of the radar?)
- Figure 3c is kind of hard to see. I would maybe remove it.
- “warm (lunch) break was essential” – I’m not sure this is necessary information.
- “The ideal number for drilling is three people” – add “for our drilling” as it’s specific to your case and equipment. Most ice core drilling require more people.
- Reference of “(Legrain *et al.*, 2025).” – please follow the reference format of the journal (numbers as superscript instead)
- “the risk of hypothermia” is mentioned many times in the report. I would say mentioning this once is enough as it’s a common problem for Antarctic fieldwork but is very easily addressable in the field in my opinion.
- “Meteorites have been found in the Nils Larsen area during the BELARE meteorite reconnaissance” – specify what the abbreviation stands for
- Re. meteorites: would it have been useful to first check datasets of meteorites (e.g., Refer to reference no. 1) and compare these to what you observed in the field? Can any information be gained from your search for meteorites that would connect with these existing satellite and ML datasets?

-------------------------------------------------------

Is the case presented with sufficient detail to be useful for teaching or other practitioners?

Yes

Is the work clearly and accurately presented and does it cite the current literature?

Yes

If applicable, is the statistical analysis and its interpretation appropriate?

Not applicable

Are all the source data underlying the results available to ensure full reproducibility?

Yes

Are the conclusions drawn adequately supported by the results?

Partly

Is the background of the case’s history and progression described in sufficient detail?

Yes

Reviewer Expertise:

Radar, Field glaciology, Climate, Ice-sheet modelling, Satellite data analysis

I confirm that I have read this submission and believe that I have an appropriate level of expertise to confirm that it is of an acceptable scientific standard, however I have significant reservations, as outlined above.

## References

[1] : Unexplored Antarctic meteorite collection sites revealed through machine learning. *Science Advances* .2022;8(4) : 10.1126/sciadv.abj8138 10.1126/sciadv.abj8138 PMC879146135080966

# Response

**Published:** 2026-05-29

**Authors:** Maaike Izeboud

**Affiliations:** Water and Climate, VUB Faculty of Engineering, Brussels, Brussels, Belgium

The manuscript is a field report, not a science paper per se. It describes the first of two field seasons in Dronning Maud Land area, aimed at recovering more information on the blue ice areas around the Princess Elizabeth Station. The report describes the key methods and equipment used and discusses the limitations (particularly weather-related) associated with each of the methods. I found it interesting to read as it is relatively rare nowadays to publish field reports which can help and guide future surveys attempting to do the same thing.

 This paper is well written, and the figures are clear for the most part. I only have a few minor comments regarding the text or figures but overall, I would recommend this paper to be indexed in Open Research Europe with minor revision.

 With best wishes,

 Dr. Julien Bodart We thank the reviewer for their positive words and respond to each of their comments below.

**General comments** **[R2-1] **I think the only thing I am missing is a conclusion section, describing the key achievements from the field season, and what has been learned for season 2. Perhaps some limitations associated with the methods or equipment used and which will be taken forward for the next field season?

 This is a good suggestion and has been implemented by adding a short conclusion section.

**Line-by-line comments:**

**[R2-2] **Abstract: Please state what the abbreviation “QUOI” stands for. Also remove “…” in “CO2, CH4, N2O ...)” everywhere in the paper and either state all or say “amongst others”.

 We have added ‘amongst others’ everywhere in the paper. Rather than detailing ‘QUOI’ in the abstract, we have removed this part of the sentence from the abstract (‘ *funded by the Belgian Science Policy Office (BELSPO), with logistical support from the International Polar Foundation (IPF) and additional funding from the QUOI project*’). The QUOI project and acronym is introduced fully in the Introduction: **Qu**est for world’s **o**ldest **i**ce.

**[R2-3] **“These products provided a preliminary understanding of the area but were too coarse and uncertain to capture local processes that are important to determine a drilling site.” – please specify this is only applicable to the use of bedmachine/bedmap2/measures, and not the AWI radar profiles used, as AWI radars have a much higher resolution that is enough to guide ice core drilling.

 Indeed, we did not mean to imply the resolution of the AWI radars. However, the available profiles did not provide us enough information to guide our ice core drilling site selection (requiring not only bedrock depth but a broader understanding of it’s surrounding), which we have clarified by adding: “ *These products provided a preliminary understanding of the area but were **too sparsely distributed**, too coarse **and/or** uncertain to capture local processes that are important to determine a drilling site*”.

**[R2-4] **“In addition, estimated terrestrial age of meteorites found on the blue ice in the Nils Larsen ...” – please provide a reference for this sentence

 The terrestrial ages are unfortunately not published, which has been added to the manuscript. A reference for the recovery of said meteorites is added: https://www.sciencedirect.com/science/article/abs/pii/S0016703718307245

**[R2-5] **“with four patches considered to have the highest priority (Patch 9, Patch 8, Patch 4 and Patch 2; Figure 1b).” – why? Explain

 Added *“due to a combination of their (low) ice flow velocity and thickness as well as suspected ice flow configuration, estimated from the data products used during pre-season planning and analysis.”*

**[R2-6] **Figure 1: it would be useful to provide at least one figure with coordinates on x and y axis.

 Good point, this has been added to Figure 1b.

**[R2-7] **“A surface ice sample transect along the direction of ice flow, sometimes two transects.” Since you state that each patch had the same set of measurements at every patch, then maybe say “At least one surface ice sample transect along the direction of ice flow”.

 Agreed, the text has been revised.

**[R2-8] **“‘fake meteorites’” – please explain

 The text is revised to clarify these are metal cilinders that we refer to as ‘fake meteorites’: *“…placed and monitored metal cylinders (‘fake meteorites’) in Patch 3 and 5 to measure…”*  

**[R2-9] **“We used a MALA RTA of 25 MHz with an antenna distance of 6 m to measure ice thickness, from which bed topography can be derived.” – this doesn’t really make sense. Bed topography is imaged, which by subtracting with surface return gives ice thickness. Also, maybe it should be bed elevation, in which case you should state that this is obtained by subtracting ice thickness from surface elevation observed with GNSS at the surface. Instead of Figure 2, I would add a table which describes the key parameters used.

 We have rephrased “We used a MALA RTA of 25 MHz with an antenna distance of 6 m to measure the ice-bed interface, from which ice thickness can be derived and bed elevation is subsequently calculated by subtracting it from the surface elevation.”

 We agree with the reviewer and have converted Figure 2 into a table (Table 1).

**[R2-10] **“To identify areas with ice thickness < 200 m and ideally a ‘cul-de-sac’ topography, used to determine the site for next field mission.” This sentence does not make sense grammatically. Also is “cul-the-sac” topography really a well-known thing? Is there an equivalent in English that would be easier for non-French speakers to understand?

 To our knowledge the ‘cul-the-sac’ is more often used to describe potential trapping locations of old ice. Nevertheless we concur this is a very specific term and have rephrased the sentence: *“We aimed to identify areas with ice thickness < 200 m and favorable bed topography for trapping or containing old ice (e.g. a closed basin, also referred to as ‘cul-de-sac’) to guide the site selection for next field mission.”*

**[R2-11] **“Moreover, initial processing was done in the basecamp (using ReflexW software14) to select suitable locations for the 10-m depth ice cores in each patch.” I would rephrase this sentence as you mention later what the processing consisted of.

 Good point, we revised to “ *In addition, after initial processing of the data at the basecamp, we used the thickness profiles to select suitable locations for the 10-m depth ice cores within each patch*”

**[R2-12] **“In-field processing” for the radar: please also state how you acquired the gps positions of each trace (GNSS station embedded on top of the radar?) Figure 3c is kind of hard to see. I would maybe remove it.

 Panel 3c has been removed.

 We have specified in the first section (before ‘objective’), where we describe the MALA system: “ *The MALA system includes a connected* *GPS receiver (GLOBALSAT BU-353S4) which immediately co-registers coordinates (latitude, longitude) and surface elevation to each datapoint”.*

**[R2-13] **“warm (lunch) break was essential” – I’m not sure this is necessary information.

 We understand this does not seem relevant. We wanted to include this as it was an important part of our day-to-day planning, and in general determined how much area we were able to cover. We have reorganized the “weather limitations” section, as it was quite repetitive (see also comment **R2-16** and **R3-1**) and have moved this information to the ‘encountered challenges’ part of the MALA section. The relevant revised text now reads:

***“*** *Duration of field activity: we initially expected to complete ~10 km of transect per day, but often reached ~20 km. On colder or windier days (> 3 bft), this was only possible when shelter was available along the route or by returning to basecamp halfway”*

**[R2-14] **“The ideal number for drilling is three people” – add “for our drilling” as it’s specific to your case and equipment. Most ice core drilling require more people.

 Agreed, revised to “Our drilling operations were typically carried out by a team of three, …”

**[R2-15] **Reference of “(Legrain *et al.*, 2025).” – please follow the reference format of the journal (numbers as superscript instead)

 Done.

**[R2-16] **“the risk of hypothermia” is mentioned many times in the report. I would say mentioning this once is enough as it’s a common problem for Antarctic fieldwork but is very easily addressable in the field in my opinion.

 Yes, we agree. This stemmed from our intent to include the same sub-section structure for every measurement activity, but resulted in repetitive info for this topic. We have removed the ‘weather limitations’ sections and bundled comments on hypothermia in the “general approach in the field” section; other remaining equipment-related weather limitations are moved to the respective ‘encountered challenges’ sections.

**[R2-17] **“Meteorites have been found in the Nils Larsen area during the BELARE meteorite reconnaissance” – specify what the abbreviation stands for

 Added, it stands for “BELgian Antarctic Research Expedition.

**[R2-18] **Re. meteorites: would it have been useful to first check datasets of meteorites (e.g., Refer to reference no. 1) and compare these to what you observed in the field? Can any information be gained from your search for meteorites that would connect with these existing satellite and ML datasets?

 Yes definitely, analysis of meteorites was done in the pre-season planning to get a first-order esitmate of the age of the blue ice areas at their surface. Gaining information from what we found in the field, however, is less clear, since we do not have enough information to determine the reason why we did not find any meteorites (were all meteorites already collected in the mission two years prior? Did the meteorites sink below the surface? Did we just not spend enough time to find them?). We have had many discussions with the ULTIMO team who were recovering meteorites in a parallel expedition, and think the potential to use their data (>100 meteorites) have higher potential to yield insights with respect to e.g. ML datasets of Tollenaar et al. (ref 28 and 29).

# Reviewer report: Reviewer response for version 1

**DOI:** 10.21956/openreseurope.23325.r70112

**Published:** 2026-03-20

**Authors:** Thomas Laepple

**Affiliations:** 1 Alfred Wegener Institute (AWI), Potsdam, Germany

This manuscript presents an expedition report of a field campaign designed to identify a suitable drilling site for a shallow ice core (up to 200 m) in the Nils Larsen blue ice area. The stated aim of the manuscript is to document the preparation and execution of the field campaign and to provide a “blueprint for future Antarctic fieldwork”.

 The manuscript is a useful contribution for other groups doing future Antarctic fieldwork. However, the clarity of the manuscript should be improved, as parts of the text are somewhat informal or imprecise. Please see the detailed comments below.

 Detailed comments: 

 Ground Penetrating Radar: MALA Rough Terrain Antenna (RTA)

 "We used a MALA RTA of 25 MHz"

 The system is not described in detail (which receiver unit / monitor), which battery (just the stock one 

 "Insights from the MALA data"

 -> (MALA is a company, not a system)

 "The drilling was performed with a Metabo b7532/4"

 -> This is just the electric motor; describe the drilling system

 "We did not directly collect the ice at the surface to exclude the layer influenced by seasonal temperature variations, which could alter the gas content and compromise the dating16 ."

 What means surface here? Seasonal temperature variations should go down to >10m depth (depending on what amplitude matters)

 10m cores, field execution:

 I found this section somewhat difficult to follow, as it does not clearly describe which samples were collected and how this differed between the different objectives. A more structured description of sampling strategy and workflow would improve clarity.

 "Pancake ice"

 The term “pancake ice” is typically used in the context of sea ice and wave-influenced environments. Its use here in an ice-coring context is potentially confusing.

 "We only consistently logged the temperature of the ice for the two full-depth cores (see Table 1), as often the drilling of the small hole for the thermometer would break the sample in smaller pieces."

 Why did you log the temperature of the ice and not of the borehole?

 "The cable of the drill head": Do you mean the electrical cable to the motor? 

 "The springs at the bottom of the barrel, which prevent the core from falling out," they are usually called core-catchers. 

 "We calibrated the blades of the drill to adapt the settings to blue ice (versus firn) as we could initially clearly see drill-lines on the side of the core samples." 

 Maybe more details here would be useful 

 "Difficulty getting the ice core out of the barrel" Did you knock on the barrel from the side; this often helps (no need to change the manuscript here)

 Surface isotope samples

 "Collect (melted) surface ice samples" Do you really want to collect melted samples? I guess you mean that they will be melted in the lab

 "Requiring removing the first few cm of ice and then taking the next few cm of ice as the actual sample." This is not very precise; how much was removed, how much was used as sample

 "(mountainor snowmobile)" what does this word mean?

 "sampling PP bottle" define PP; better describe once what bottle type (manufacturer, size) you are using

 "with (handheld) GPS" grammar

 “field administration” is somewhat unclear; consider replacing it with “field handling” or “sample tracking” for clarity.

 "For the approach with the ice screw, it is important to have high quality screws (and including a free rotating handle; e.g., petzl screws)." Please detail which one you used.

 "We deployed a Phase-sensitive radar (ApRES) on two locations on each blue ice patch" Again be more detailed about the device and setup

 "Ideally the ApRES measurements are performed on ice thicker than 200 meters as the resolution of the instrument is limited by its widely tested standard settings (other than the calibration parameters that need to be changed). We did not aim to change these settings, as this would not guarantee good results. The choice of location was therefore a trade-off between the distance to the ice core and the thickness of the ice. The location choice was made after analysing initial MALA results. Most of the measurements were done with the same calibration settings, and the data was processed in the camp for an initial quality check. At four locations, data quality was deemed to be insufficient. After recalibration, these four sites were remeasured using different calibration settings."

 This whole paragraph is not very informative; Changing the standard settings does not guarantee good results, 'most' of the measurements... 

 "quand-polarimetric configuration"  is this the right term?

 "The battery of the ApRES (a snowmobile battery)" Be more detailed; Pb or Lithium, capacity..

 " a multitude of randomly distributed ablation stakes" type + not very precise. How many are a multitude.

 “a measurement subject to hypothermia risks” is misleading, as the risk applies to the operators rather than the measurement itself.

 "We always connected a snowmobile-battery to the system." Again, if the challenge is the battery, please specify it's exact type to be useful for the reader

Is the case presented with sufficient detail to be useful for teaching or other practitioners?

Partly

Is the work clearly and accurately presented and does it cite the current literature?

Partly

If applicable, is the statistical analysis and its interpretation appropriate?

Not applicable

Are all the source data underlying the results available to ensure full reproducibility?

Yes

Are the conclusions drawn adequately supported by the results?

Yes

Is the background of the case’s history and progression described in sufficient detail?

Yes

Reviewer Expertise:

Antarctic field work, ice-coring, climate research, statistics, climate variability, oxygen isotopes

I confirm that I have read this submission and believe that I have an appropriate level of expertise to confirm that it is of an acceptable scientific standard, however I have significant reservations, as outlined above.

# Response

**Published:** 2026-05-29

**Authors:** Maaike Izeboud

**Affiliations:** Water and Climate, VUB Faculty of Engineering, Brussels, Brussels, Belgium

This manuscript presents an expedition report of a field campaign designed to identify a suitable drilling site for a shallow ice core (up to 200 m) in the Nils Larsen blue ice area. The stated aim of the manuscript is to document the preparation and execution of the field campaign and to provide a “blueprint for future Antarctic fieldwork”.

 The manuscript is a useful contribution for other groups doing future Antarctic fieldwork. However, the clarity of the manuscript should be improved, as parts of the text are somewhat informal or imprecise. Please see the detailed comments below.

 We thank the reviewer for their positive words and their comments. We have implemented their suggestions, which is detailed in responses to each comment below, and made additional effort throughout the text to rephrase text less informal.

**Detailed comments:** 

**[R1-1]** Ground Penetrating Radar: MALA Rough Terrain Antenna (RTA)

 "We used a MALA RTA of 25 MHz".

 The system is not described in detail (which receiver unit / monitor), which battery (just the stock one 

**Response**: Figure 2 has been converted into a table where more system detailes are included: *Antenna: MALÅ Rough Terrain Antenna 25mHz*

*Receiver unit: MALÅ Professional Explorer (ProEx) control unit, Li-Ion 12 V battery.*

*Monitor: MALÅ XV Monitor*

**[R1-2]** "Insights from the MALA data"

 -> (MALA is a company, not a system)

**Response**: The text has been chanced to “MALA GPR data”

**[R1-3]** "The drilling was performed with a Metabo b7532/4"

 -> This is just the electric motor; describe the drilling system

**Response**: A short description is added: “ *The core barrel was* *handcrafted by the technical team at the Université libre de Bruxelles, along with the cutting blades used to drill the ice, and had an outer diameter of 8 mm and was made of stainless steel*.”

**[R1-4]** "We did not directly collect the ice at the surface to exclude the layer influenced by seasonal temperature variations, which could alter the gas content and compromise the dating16 ." What means surface here? Seasonal temperature variations should go down to >10m depth (depending on what amplitude matters)

**Response**: The text has been adjusted to state “ *we did not collect the ice of the first seven meters*” to clarify which samples were excluded and nuance has been made to emphasize the influence of surface processes on gas content.

**[R1-5]** 10m cores, field execution: I found this section somewhat difficult to follow, as it does not clearly describe which samples were collected and how this differed between the different objectives. A more structured description of sampling strategy and workflow would improve clarity.

**Response**: The structure of the section has been altered and divided into three paragraphs, (1) detailing practical info on duration and number of teammembers (2) objective 1 sampling (3) objective 2 sampling. Text in this section has been strongly rewritten to improve clarity of descriptions.

**[R1-6]** "Pancake ice". The term “pancake ice” is typically used in the context of sea ice and wave-influenced environments. Its use here in an ice-coring context is potentially confusing. **Response**: Agreed this could potentially be confusing. We have removed this term from the text.

**[R1-7]** "We only consistently logged the temperature of the ice for the two full-depth cores (see Table 1), as often the drilling of the small hole for the thermometer would break the sample in smaller pieces." Why did you log the temperature of the ice and not of the borehole?

**Response**: It is easier in term of materials to log the core itself rather than the borehole. This measure was just to get a rough idea of the ice temperature, so measuring the ice temperature was ok.

**[R1-8]** "The cable of the drill head": Do you mean the electrical cable to the motor? **Response**: Yes. This has been added to the respective sentence.

**[R1-9]** "The springs at the bottom of the barrel, which prevent the core from falling out," they are usually called core-catchers. 

**Response**: This term has been added to the text.

**[R1-10]** "We calibrated the blades of the drill to adapt the settings to blue ice (versus firn) as we could initially clearly see drill-lines on the side of the core samples." 

 Maybe more details here would be useful **Response**: We added: *“* *The optimal setting was 1.0 mm (the blades protrude 1.0 mm from the top of the core barrel)”*

**[R1-11]** "Difficulty getting the ice core out of the barrel" Did you knock on the barrel from the side; this often helps (no need to change the manuscript here) **Response**: Yes, although due to the often damaged conditions of the ice this method was not favored as we did not want to promote further fracturing of the ice.

**Surface isotope samples**

**[R1-12]** "Collect (melted) surface ice samples" Do you really want to collect melted samples? I guess you mean that they will be melted in the lab **Response**: We chose to melt them in our basecamp, mainly for logistic reasons. For the intendend measurements the important aspect was that the samples did not undergo multiple melt-freeze cycles (i.e. we should only melt them once). However, this nuance is explained at a later section of the text and is not relevant here, so we have removed the ‘(melted)’ in this sentence.  

**[R1-13]** "Requiring removing the first few cm of ice and then taking the next few cm of ice as the actual sample." This is not very precise; how much was removed, how much was used as sample

**Response**: This has been clarified to “ *the ice is taken below the uppermost surface layer by removing the first few cm (approximately 8-10 cm) of ice and then taking the next 8-10 cm of ice as the actual sample*”.

**[R1-14]** "(mountainor snowmobile)" what does this word mean?

**Response**: This has been clarified to “using far-ahead reference points (e.g. a mountain top or parked snowmobile)”.

**[R1-15]** "sampling PP bottle" define PP; better describe once what bottle type (manufacturer, size) you are using

**Response**:  It’s a polypropylene (PP) bottle; the sentence has been revised, see next comment (R1-16).

**[R1-16]** "with (handheld) GPS" grammar

**Response**:  The sentence has been revised to: *“A second person followed, prepared the polypropylene (PP) bottle to deposit the ice sample (60 ml, 33 mm diameter, 70 mm height, brand:* *VWR ^®^), kept track of the correct bottle number for each sample, and marked the sample location with a handheld GPS.”*

**[R1-17]** “field administration” is somewhat unclear; consider replacing it with “field handling” or “sample tracking” for clarity.

**Response**:  Good suggestions; we replaced with ‘field handling’.

**[R1-18]** "For the approach with the ice screw, it is important to have high quality screws (and including a free rotating handle; e.g., petzl screws)." Please detail which one you used.

**Response**: We meant to imply we used Petzl screws. Rephrased sentence to: “ *We used high-quality ice screws with a free-rotating handle (Petzl screws), which proved essential for efficient sampling*”

**[R1-19]** "We deployed a Phase-sensitive radar (ApRES) on two locations on each blue ice patch" Again be more detailed about the device and setup

**Response**: We have added specification: ApRES phase-sensitive radar is a low-power, light-weight instrument developed in a collaboration between the British Antarctic Survey and University College London. It is a 200-400 MHz FMCW radar, with a 1-second chirp (nominal power of 20dBm, or 100 mW), run by controller in yellow Pelican Box, powered by a snowmobile battery (Pb, 12 V/18 Ah). Detail about the set-up is included in the ApRES template (supplementary material), of which an example is shown in Figure 9.

**[R1-20]** "Ideally the ApRES measurements are performed on ice thicker than 200 meters as the resolution of the instrument is limited by its widely tested standard settings (other than the calibration parameters that need to be changed) We did not aim to change these settings, as this would not guarantee good results. The choice of location was therefore a trade-off between the distance to the ice core and the thickness of the ice. The location choice was made after analysing initial MALA results. Most of the measurements were done with the same calibration settings, and the data was processed in the camp for an initial quality check. At four locations, data quality was deemed to be insufficient. After recalibration, these four sites were remeasured using different calibration settings."

 This whole paragraph is not very informative; Changing the standard settings does not guarantee good results, 'most' of the measurements... 

**Response**:  We concur this was vaguely worded, and contains irrelevant information. The section has been rewritten and split into two pagraphs: *“We deployed a Phase-sensitive radar (ApRES) on two locations on each blue ice patch (Figure 1). In some cases, these locations coincided with the ice core site, while in others they were selected for their added value in providing bedrock information. ApRES measurements are ideally conducted on ice thicker than 200 meters to ensure reliable signal returns. The choice of location was therefore a trade-off between the distance to the ice core and the thickness of the ice, which was informed by preliminary MALA radar results.* *We used consistent calibration settings across most sites and performed initial data processing in the camp for a data quality assessment. At four locations, data quality was insufficient, and the these four sites were subsequently remeasured with different settings after recalibration (detailed below).”*

**[R1-21]** "quand-polarimetric configuration"  is this the right term?

**Response**: This contains a typo, it should be **quad**-polarimetric configuration.

**[R1-22]** "The battery of the ApRES (a snowmobile battery)" Be more detailed; Pb or Lithium, capacity..

**Response**: The specifics have been added (see comment R1-19).

**[R1-23]** " a multitude of randomly distributed ablation stakes" type + not very precise. How many are a multitude.

**Response**: We have replaced this with the actual number of distributed stakes: 69.

**[R1-24]** “a measurement subject to hypothermia risks” is misleading, as the risk applies to the operators rather than the measurement itself.

**Response**: The reviewer is correct. We have removed the ‘weather limitations’ sections and bundled comments on hypothermia in the “general approach in the field” section (see also comment **R2-16** and **R3-1**). The relevant text in the general section reads: *“In particular, ApRES and GPR measurements were postponed on days with wind speeds of 3–4 bft, as these activities require operators to remain exposed and stationary for extended periods. Under such conditions, we instead prioritized activities less sensitive to wind exposure, including drilling (with shelter), surface water isotope sampling, and meteorite searches. GNSS measurements were performed together with ApRES measurements and therefore postponed depending on ApRES limitations.”* Other remaining equipment-related weather limitations are moved to the respective ‘encountered challenges’ sections.

**[R1-25]** "We always connected a snowmobile-battery to the system." Again, if the challenge is the battery, please specify it's exact type to be useful for the reader

 Done.
